# Trimester effects of source-specific PM_10_ on birth weight outcomes in the Avon Longitudinal Study of Parents and Children (ALSPAC)

**DOI:** 10.1186/s12940-020-00684-w

**Published:** 2021-01-07

**Authors:** Yingxin Chen, Susan Hodgson, John Gulliver, Raquel Granell, A. John Henderson, Yutong Cai, Anna L. Hansell

**Affiliations:** 1 Centre for Environmental Health and Sustainability, George Davies Centre, University of Leicester, University Road, Leicester, LE1 7RH UK; 2 MRC-PHE Centre for Environment and Health, Department of Epidemiology and Biostatistics, School of Public Health, Imperial College London, London, UK; 3 MRC Integrative Epidemiology Unit, Population Health Sciences, Bristol Medical School, University of Bristol, Bristol, UK; 4 Nuffield Department of Women’s and Reproductive Health, University of Oxford, Oxford, UK; 5 Deep Medicine Programme, Oxford Martin School, University of Oxford, Oxford, UK

**Keywords:** Air pollution, Environmental health, Epidemiology, Dispersion modeling, Particulate matter, Birth weight, Preterm birth, ALSPAC

## Abstract

**Background:**

Evidence suggests that exposure to particulate matter with aerodynamic diameter less than 10 μm (PM_10_) is associated with reduced birth weight, but information is limited on the sources of PM_10_ and exposure misclassification from assigning exposures to place of residence at birth.

**Methods:**

Trimester and source-specific PM_10_ exposures (PM_10_ from road source, local non-road source, and total source) in pregnancy were estimated using dispersion models and a full maternal residential history for 12,020 births from the Avon longitudinal study of parents and children (ALSPAC) cohort in 1990–1992 in the Bristol area. Information on birth outcomes were obtained from birth records. Maternal sociodemographic and lifestyle factors were obtained from questionnaires. We used linear regression models for continuous outcomes (birth weight, head circumference (HC), and birth length (BL) and logistic regression models for binary outcomes (preterm birth (PTB), term low birth weight (TLBW) and small for gestational age (SGA)). Sensitivity analysis was performed using multiple imputation for missing covariate data.

**Results:**

After adjustment, interquartile range increases in source specific PM_10_ from traffic were associated with 17 to 18% increased odds of TLBW in all pregnancy periods. We also found odds of TLBW increased by 40% (OR: 1.40, 95%CI: 1.12, 1.75) and odds of SGA increased by 18% (OR: 1.18, 95%CI: 1.05, 1.32) per IQR (6.54 μg/m^3^) increase of total PM_10_ exposure in the third trimester.

**Conclusion:**

This study adds to evidence that maternal PM_10_ exposures affect birth weight, with particular concern in relation to exposures to PM_10_ from road transport sources; results for total PM_10_ suggest greatest effect in the third trimester. Effect size estimates relate to exposures in the 1990s and are higher than those for recent studies – this may relate to reduced exposure misclassification through use of full residential history information, changes in air pollution toxicity over time and/or residual confounding.

**Supplementary Information:**

The online version contains supplementary material available at 10.1186/s12940-020-00684-w.

## Introduction

Maternal exposure to particulate matter (PM) may affect fetal growth, resulting in adverse birth outcomes, including infant death, stillbirth, preterm birth (PTB), term low birth weight (TLBW) and small for gestational age (SGA) [1, 2]. This is of public health importance as adverse birth outcomes have been consistently associated with increased risk of chronic conditions in later adulthood such as obesity [3], diabetes [3, 4], and cardiovascular diseases (CVDs) [5].

There are several studies that have investigated effects of PM_10_ by trimester, but results regarding potential sensitive time windows during pregnancy on adverse birth outcomes are inconsistent [6–11]. Reviews and meta-analyses [2, 12–16] have reported 1st trimester or 3rd trimester as a possible critical window of exposure for PTB. An important source of bias in most of the epidemiological studies to date is exposure misclassification related to assigning exposure to the maternal residential location at the time of birth. Using exposure estimates based on address at birth may lead to a higher exposure misclassification for the 1st trimester than for the 3rd trimester. The duration of the third trimester is seldom taken into account in studies of trimester-specific effects of ambient air pollution exposures; it may be important for exposures whose composition and concentration varies over time. Additionally, while some studies have investigated PM_2.5_ sources or chemical components in relation to pregnancy outcomes [17–22], fewer studies have considered sources of PM_10_ [10, 23–25].

This study used the Avon Longitudinal Study of Parents and Children (ALSPAC), with source-specific maternal air pollution exposure derived from dispersion models that distinguished between local and regional sources for all maternal residential addresses in pregnancy. The aim of this analysis was to investigate the associations between source-specific air pollution in each trimester of pregnancy and adverse birth outcomes, including birth weight, PTB, TLBW, SGA, head circumference (HC), and birth length (BL).

## Methods

### Study design and population

**Fig. 1:**
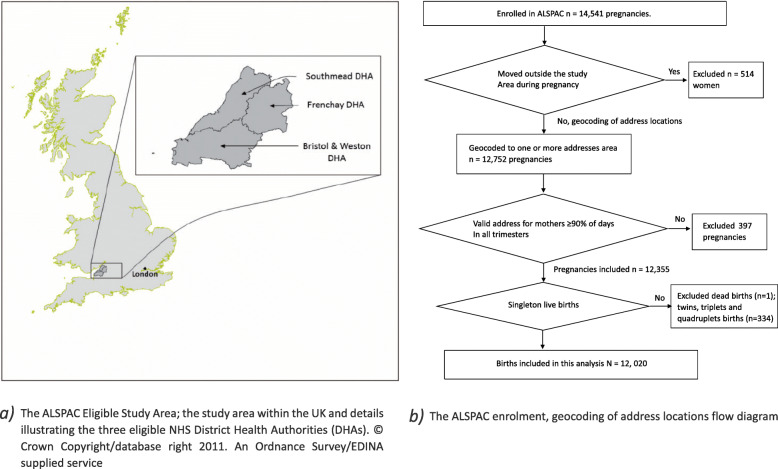
The ALSPAC enrolment and geocoding of address locations flow diagram [26, 27]. **a** The ALSPAC Eligible Study Area; the study area within the UK and details illustrating the three eligible NHS District Health Authorities (DHAs).© Crown Copyright/database right 2011. An Ordnance Survey/EDINA supplied service. **b** The ALSPAC enrolment, geocoding of address locations flow diagram.

The ALSPAC cohort is a population-based cohort study located in south-west England (Fig. 1 a) [26, 28, 29]. The study area covered the three health administration districts, includes the City of Bristol (1991 population ∼0.5 million) and surrounding urban and rural areas, including towns, villages, and farming communities.

All pregnant women who lived in this area with an expected delivery date between 1 April 1991 and 31 December 1992 were eligible for the study [26]. In total, the initial number of pregnancies enrolled in ALSPAC was 14,541, among these 657 women participating had more than one pregnancy. Analyses were restricted to mothers who had valid addresses for at least 90% of days in each trimester (Fig. 1 b). Twins, triplets, and quadruplets were excluded. A total of 12,020 live births were included in this analysis.

Ethical approval for this study was obtained from the ALSPAC Ethics and Law Committee and Local Research Ethics Committees.

### Exposure assessment

A detailed description of the exposure assessment is presented in the paper of Gulliver et al. [30]. In brief, we used dispersion models for specific sources of local traffic, local industry and long-range particulates. We used the ADMS-Urban model to estimate local traffic and non-traffic daily PM_10_ (referred to as PM_10__road and PM_10__other hereafter) within the study area. PM_10__road was modelled from each main road, which have annual average daily traffic (AADT) > 5000, as a line source. PM_10__other was modelled using meteorological data such as hourly values of wind speed, wind direction, cloud cover, and temperature obtained from British Atmospheric Data Centre (BADC) (www.badc.ac.uk) and information available on a 1 km grid from the National Atmospheric Emissions Inventory (NAEI) (http://naei.defra.gov.uk/data/mapping).

The NAME-III air pollution model (Numerical Atmospheric-dispersion Modelling Environment) was used to estimate regional/long-range sources (i.e. outside the study area) of daily PM_10_ using meteorological data taken from the ERA-Interim meteorological reanalysis produced by European Centre for Medium-Range Weather Forecasts (ECMWF) and pollution data in the form of daily average concentrations of PM_10_ for 14 receptor locations. Total short-term PM_10_ (referred to as PM_10__total hereafter), were evaluated using Eq. (1) below [30]. A constant, labelled NATURAL in Eq. (1), of 12.0 μg/m^3^ was included in the model to account for local non-anthropogenic sources of PM_10_ (e.g. wind-blown soil and other crustal matter).

1 $$ {\mathrm{PM}}_{10}\_\mathrm{total}=\mathrm{ADMS}-\mathrm{Urban}\ \left({\mathrm{PM}}_{10}\_\mathrm{road}\ \mathrm{and}\ {\mathrm{PM}}_{10}\_\mathrm{other}\right)+\mathrm{NAME}-\mathrm{III}+\mathrm{NATURAL} $$

For the period of our study, monitoring in the Bristol area varied, but remained limited: from 1990 to 1992 there was no PM_10_ air pollution monitoring in the area; from January 1993 until September 2005 there was a single site. In the absence of enough monitoring data to evaluate the model for periods relating to pregnancy trimesters, we validated the model using weekly averaged measured concentrations from 1993 [30].

We assigned exposure estimates based on home address locations and averaged address-time-weighted PM_10_ exposure estimates in each trimester and the whole pregnancy (WP) [27, 31]. The date of conception was estimated from 2 weeks after the last menstrual period (LMP). We defined trimester 1 (T1) as spanning from the date of conception to day 91 of pregnancy, trimester 2 (T2) as days 92–183 and trimester 3 (T3) as days 184 to delivery (if delivery took place after day 184).

### Adverse birth outcomes

Main outcome measures were derived by abstraction of maternal medical records by trained research midwives/nurses and included birth weight (in grams, continuous), HC (cm, continuous), BL (cm, continuous), PTB (binary), TLBW (binary), and SGA (binary) [29]. PTB was defined as a live birth delivered at more than 22 weeks’ and less than 37 weeks’ gestation. TLBW was defined as weighing less than 2500 g at birth after 37 weeks of gestation. SGA was subsequently defined as birth weight z-score below the 10th percentile, where birth weight for gestational age by sex (birth weight z-score) was calculated using a recently updated birth weight reference based on UK data from the same period (early 1990s) [32].

### Confounders

The following potential confounders were identified a priori: infant’s sex (male/female), maternal age at 8 weeks of gestation (in years), pre-pregnancy maternal BMI (BMI < 18.5/18.5 ≤ BMI < 25/25 ≤ BMI < 30/BMI ≥ 30, kg/m^2^), maternal educational level (Low: O level or below/High: A level or above), maternal smoking during first 3 months of pregnancy (Yes/no), and environmental tobacco smoke exposure during pregnancy (Yes/no).

Information on confounders was collected from self-completed questionnaires from mothers at 8 weeks, 12 weeks, 18 weeks and 32 weeks of pregnancy [28]. Informed consent for the use of data collected via questionnaires and clinics was obtained from participants following the recommendations of the ALSPAC Ethics and Law Committee at the time.

### Statistical analyses

Modelled source-specific PM_10_ exposure levels in T1, T2, T3, and WP were considered separately. We checked the correlation between each of the exposures in the different pregnancy periods. We also checked the correlations between PM_10__road and PM_10__other in the same pregnancy period. Multivariable linear regression was used for continuous birth outcomes and multivariable logistic regression for binary outcomes. Effect estimates are reported for each IQR increase of PM_10_.

We also tested for effect modification by sex, maternal education and gestational age of the associations between PM for all pregnancy period and the birth outcomes Effect modification by ethnicity was not computed in this study because only 2.2% of the 80% of women who answered the question at 8 months post-birth identified themselves as non-white ethnicity, representative of the area at that time. We also ran mutually-adjusted models to estimate associations of one PM_10__total trimester-average exposure (TAE) with birth outcomes, jointly adjusted for the other two PM_10__total TAEs (i.e. adjusted PM_10__total in T2 and T3 for PM_10__total in T1). We additionally conducted some multiply adjusted analyses to fit PM_10_ road and PM_10_ other simultaneously to the models for each pregnancy period.

### Sensitivity analysis

The length of pregnancy may affect our results. Therefore, for the TLBW and SGA analyses, we compared whole pregnancy exposures truncated to 37 weeks among term births to ensure equal length of exposures.

Given the proportion of missing data on confounder variables were high in the study population, analyses based on complete cases (i.e. those individuals who have no missing data in any of the variables required for that analysis) may be biased. Therefore, we used multiple imputation (MI) analysis [33, 34]. We used the multivariate normal (MVN) iterative method for imputation using Stata’s *mi impute mvn* command. The following variables were imputed: maternal age, pre-pregnancy maternal BMI, maternal educational level, maternal smoking, and environmental tobacco smoke exposure during pregnancy. Exposure and outcome variable of each models were considered as observed covariates and used in the models to impute theses variables. For each imputation model, 10 imputations were run. We then fitted the desired model separately on each of the 10 imputed datasets and combine the results for all regression models.

All analyses were performed using STATA version 15.

## Results

Table 1 shows that 5.00, 1.90 and 7.72% of the 12,020 births considered in the analysis were classified as PTB, TLBW and SGA, respectively.

**Table 1:** Characteristics of study sample in the Avon Longitudinal Study of Parents And Children cohort (*N* = 12,020)

Characteristic of study births	N^***a***^	n (%) or mean ± SD	Characteristic of mothers	N^***a***^	n (%) or mean ± SD
Completed gestational weeks	12,020	39.48 ± 1.83	Maternal age (Years)	11,250	27.79 ± 4.9
Birth weight (g)	11,896	3415.42 ± 540.13	Height (Cm)	10,534	163.92 ± 6.72
Head circumference (Cm)	9245	34.80 ± 1.50	Pre-pregnancy weight (Kg)	10,054	61.65 ± 10.93
Birth length (Cm)	7583	50.59 ± 2.14	Maternal BMI (Kg/m^2^)	12,020	
Infant’s sex	12,020		BMI < 18.5		510 (4.24%)
Male		6215 (51.71%)	18.5 ≤ BMI < 25		7359 (61.22%)
Female		5805 (48.29%)	25 ≤ BMI < 30		1537 (12.79%)
Unknown		0	BMI ≥ 30		552 (4.59%)
PTB	12,020		Unknown		2062 (17.15%)
PTB		601 (5.00%)	Race	12,020	
Not PTB		11,419 (95.00%)	White		10,361 (86.20%)
Unknown		0	Non-White^***b***^		276 (2.30%)
TLBW	11,419		Unknown		1383 (11.51%)
TLBW		217 (1.90%)	Maternal education level	12,020	
Not TLBW		11,085 (97.08%)	Low: O level or below		6986 (58.12%)
Unknown		117 (1.02%)	High: A level or degree		3729 (31.02%)
SGA	12,020		Unknown		1305 (10.86%)
SGA		928 (7.72%)	Maternal smoke	12,020	
Not SGA		10,968 (91.25%)	Non-smoker		8538 (71.03%)
Unknown		124 (1.03%)	Smoker		2790 (23.21%)
			Unknown		692 (5.76%)
40 births are both PTB & SGA			Passive smoke	12,020	
			Not exposed		3516 (29.25%)
			Exposed		5543 (46.11%)
			Unknown		2961 (24.63%)

^***a***^represents number of births included in analysis

^***b***^Non-white population including Black Caribbean, Black African, other black, Indian, Pakistani, Bangladeshi, Chinese and others

**Fig. 2:**
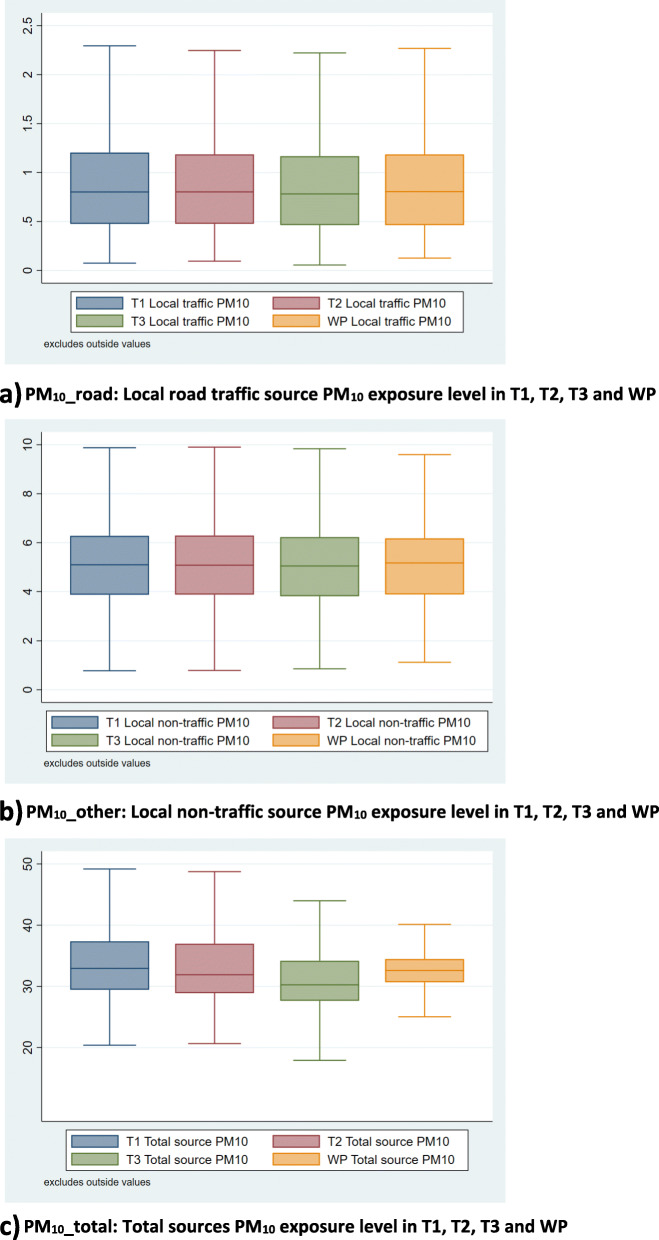
Distributions of source-specific PM_10_ exposure level in each trimester (T1, T2, and T3) and combined across whole pregnancy period (WP) (a, b, c) (n = 12,020). **a** PM_10__road: Local road traffic source PM_10_ exposure level in T1, T2, T3 and WP. **b** PM_10__other: Local non-traffic source PM_10_ exposure level in T1, T2, T3 and WP. **c** PM_10__total: Total sources PM_10_ exposure level in T1, T2, T3 and WP

Median PM_10_ exposure levels were 0.81 μg/m^3^ (IQR: 0.72) for PM_10__road, 5.17 μg/m^3^ (IQR: 2.29) for PM_10__other and 32.60 μg/m^3^ (IQR: 3.79) for PM_10__total during the whole pregnancy period (Fig. 2**,** see eTable 1 in the Supplement). Correlations were low for between-trimester PM_10__total (Pearson’s r < 0.1) (Table 2). However, between-trimester PM_10__road values were highly correlated (Pearson’s r > 0.9). High correlations were also seen between trimesters for other sources (PM_10__other) (Pearson’s r > 0.8). Correlations for PM_10__road and PM_10__other in the same pregnancy period were not highly correlated (Pearson’s r < 0.6, see eTable 2 in the Supplement).

**Table 2:** Pearson’s correlations of different source PM_10_ between each of the four different pregnancy time periods (*N* = 12,020)

a) Correlation of PM_10__road in T1, T2, T3 and WP					b) Correlation of PM_10__other in T1, T2, T3 and WP					c) Correlation of PM_10__total in T1, T2, T3 and WP				
PM_10__road	T1	T2	T3	WP	PM_10__other	T1	T2	T3	WP	PM_10__total	T1	T2	T3	WP
T1	1				T1	1				T1	1			
T2	0.94	1			T2	0.86	1			T2	−0.03	1		
T3	0.91	0.92	1		T3	0.84	0.85	1		T3	−0.08	0.03	1	
WP	0.97	0.98	0.97	1	WP	0.95	0.95	0.94	1	WP	0.53	0.61	0.54	1

T1: first trimester; T2: second trimester; T3: third trimester; *WP* entire pregnancy period

In adjusted models (Table 3) expressed per IQR increase in exposure, PM_10__total exposure in T3 was associated with a higher risk of TLBW (OR: 1.40, 95% CI: 1.12, 1.75) and SGA (OR: 1.18, 95% CI: 1.05, 1.32),. A small decreased risk between PTB and PM_10__total was found in T2 (OR: 0.84, 95% CI: 0.71,0.99). PM_10__total exposure in T1 was found to be associated with very small increases in HC (0.10 cm, 95% CI: 0.04, 0.15) and BL (0.15 cm, 95% CI: 0.06, 0.23) after adjustment.

**Table 3:** Associations between source-specific PM_10_ exposure in different pregnancy periods and adverse birth outcomes (Fully adjusted Model)

Per IQR increase	Mean difference			Odd ratios		
	Birth weight, grams	HC, cm	BL, cm	PTB^***a***^ (case: 601)	TLBW^***b***^ (case: 271)	SGA^***c***^ (case: 928)
in exposure	(*N* = 7683)	(*N* = 6127)	(*N* = 7583)	(*N* = 7761)	(*N* = 7350)	(*N* = 7683)
**PM**_**10**_**_road (μg/m3)**						
First trimester	3.54 (−8.22, 15.31)	0.01 (−0.03, 0.05)	0.04 (− 0.02, 0.10)	1.00 (0.89, 1.12)	1.15 (0.99, 1.34)	1.02 (0.93, 1.11)
Second trimester	1.02 (−10.48,12.53)	−0.02 (−0.05, 0.02)	0.02 (− 0.04, 0.07)	1.00 (0.89, 1.11)	**1.18 (1.02, 1.37)**	1.04 (0.95, 1.13)
Third trimester	1.85 (−9.73, 13.43)	0.00 (−0.04, 0.03)	0.04 (− 0.02, 0.10)	0.99 (0.88,1.10)	**1.17 (1.00,1.36)**	1.04 (0.95,1.13)
Total pregnancy	2.06 (−10.00, 14.12)	0.00 (−0.04, 0.04)	0.04 (−0.03, 0.10)	1.00 (0.89, 1.12)	**1.18 (1.04, 1.38)**	1.03 (0.95, 1.13)
**PM**_**10**_**_other (μg/m3)**						
First trimester	13.78 (−0.14, 27.70)	**0.05 (0.07, 0.10)**	**0.10 (0.03, 0.17)**	0.97 (0.85, 1.12)	1.02 (0.82, 1.28)	1.00 (0.89, 1.12)
Second trimester	9.20 (−4.70, 23.09)	0.00 (−0.04, 0.05)	0.05 (−0.02, 0.12)	0.99 (0.86,1.13)	1.16 (0.94, 1.44)	1.05 (0.94, 1.17)
Third trimester	9.17 (−5.08, 23.43)	0.03 (−0.02, 0.07)	0.07 (−0.01, 0.14)	1.03 (0.90,1.19)	1.20 (0.97, 1.50)	1.08 (0.97, 1.21)
Total pregnancy	11.05 (−2.98, 25.09)	0.03 (−0.02, 0.07)	**0.08 (0.01, 0.15)**	1.00 (0.87, 1.15)	1.13 (0.91, 1.41)	1.05 (0.94, 1.17)
**PM**_**10**_**_total (μg/m3)**						
First trimester	8.81 (−7.85, 25.46)	**0.10 (0.04, 0.15)**	**0.15 (0.06, 0.23)**	0.97 (0.82,1.14)	0.77 (0.58, 1.01)	0.90 (0.79, 1.03)
Second trimester	5.56 (−23.71, 5.31)	−0.03 (−0.08, 0.02)	0.03 (−0.06, 0.12)	**0.84 (0.71,0.99)**	1.08 (0.83,1.41)	1.00 (0.88, 1.14)
Third trimester	1.09 (−13.54, 15.72)	0.01 (−0.04, 0.05)	0.02 (−0.05, 0.09)	1.08 (0.94, 1.24)	**1.40 (1.12, 1.75)**	**1.18 (1.05, 1.32)**
Total pregnancy	0.89 (−13.61,15.38)	0.03 (−0.02,0.07)	**0.10 (0.03, 0.18)**	1.01 (0.87, 1.16)	1.11 (0.88, 1.40)	1.05 (0.93,1.17)

Model adjusted for infant’s sex, maternal age, pre-pregnancy maternal body mass index (BMI), maternal educational level, maternal smoking during first 3 months of pregnancy, and environmental tobacco smoke exposure during pregnancy.

^***a***^represents the increase in odds of PTB for a per IQR increase in PM_10_ exposure;

^***b***^represents the increase in odds of TLBW for a per IQR increase in PM_10_ exposure;

^***c***^represents the increase in odds of SGA for a per IQR increase in PM_10_ exposure

For road traffic source air pollution, we found PM_10__road was associated with increased risk of TLBW in all pregnancy periods except T1.

We also found PM_10__other exposure in T1 was associated with very small increases in HC (0.05, 95% CI: 0.07, 0.10) and BL (0.08, 95% CI: 0.01, 0.15) after adjustment.

We observed no evidence of associations with overall birth weight. We found no evidence of interactions between exposure to any air pollutant with either sex or maternal age groups for any exposure periods but some evidence for interactions between maternal education and PM_10__other in relation to birth weight but not TLBW. For an IQR increase in PM_10__other in T3, associations for O level or below (lower level of education were − 6.52 g (95% CI: −25.65, 12.61); for A level or above (higher level of education): 28.84 g (95% CI:7.91, 49.76). For PM_10__other in WP corresponding associations for O level or below were − 0.74 g, 95% CI: −19.78, 4.09; A level or above: 25.14 g, 95% CI:4.62, 45.65birth weight. We also found an interaction effect between maternal education and PM_10__road in T1, T2, T3 and WP for HC (for O level or below: range from −0.03 to 0.00; A level or above: range from 0.11 to 0.13) (eTable 3a, eTable 3b in the Supplement) but not for other outcomes.

In PM_10__total models, co-adjustment for PM_10__total exposures in the other two trimesters (eTable 4 in the Supplement) produced little or no change to associations of PM_10__total in T3 on TLBW or SGA, or of PM_10__total in T1 on HC and BL, as noted above. We did not co-adjust for exposure in other trimesters for PM_10__road and PM_10__other because these values had been found to be highly correlated across trimesters. In multiply adjusted models for PM_10__road and PM_10__other, we found little change on effects of PM_10__other in T1 on HC (0.06 cm, 95% CI: 0.02, 0.11) and on BL (0.10 cm, 95% CI: 0.02, 0.18) (eTable 5 in the Supplement). However, results for PM_10__road in T1 on TLBW changed strengthened slightly after co-adjustment for PM_10__other and became formally statistically significant (OR: 1.19, 95% CI: 1.00, 1.42).

When we restricted whole-pregnancy exposures to 37 weeks to ensure equal length for TLBW and SGA, the results did not change more than minimally (eTable 6 in the Supplement). After adjustment, PM_10__total exposure in T3 was associated with a higher risk of TLBW and SGA (OR: 1.20, 95%CI: 1.10, 1. 31; OR: 1.21, 95%CI: 1.08, 1.36, respectively). After restricting the analysis to first child, we found higher effect sizes for all PM measures with birth weight and these became formally statistically significant for PM_10__other in T1 and WP. However, little change was seen in other outcomes. (eTable 7 in the Supplement).

In multiple imputation models (Table 4), adverse associations between air pollutants and birth weight outcomes became more prominent. We found slightly reduced effect size but narrower confidence intervals for PM_10__total and TLBW and SGA in T3. Adverse associations between PM_10__total and birth weight in WP strengthened slightly and became formally statistically significant (−16.05 g, 95% CI: −28.21, −3.88 per IQR increase). We also observed stronger associations between PM_10__road and TLBW in T1 that now reached statistical significance (OR: 1.18, 95%CI: 1.05, 1. 32). Associations of PM_10__road for T2, T3 and WP changed slightly with narrower confidence intervals. PM_10__other effects on TLBW were found in all pregnancy periods except T1 (with 19% to 23% increased odds).

**Table 4:** Associations between source-specific PM_10_ exposure in different pregnancy periods and adverse birth outcomes (Multiple imputation, fully adjusted Model)

Per IQR increase	Mean difference			Odd ratios		
	Birth weight, grams	HC, cm	BL, cm	PTB^***a***^ (case: 601)	TLBW^***b***^ (case: 271)	SGA^***c***^ (case: 928)
in exposure	(*N* = 11,896)	(*N* = 9245)	(*N* = 7583)	(*N* = 12,020)	(*N* = 11,302)	(*N* = 11,896)
**PM**_**10**_**_road (μg/m3)**						
First trimester	−4.25(−14.06, 5.55)	0.00 (−0.03, 0.03)	0.03 (−0.02, 0.08)	1.00 (0.92, 1.09)	**1.18 (1.05, 1.32)**	1.03 (0.97, 1.10)
Second trimester	−7.51(−17.02, 1.99)	−0.02 (−0.05, 0.01)	0.01 (−0.04, 0.05)	1.01 (0.93, 1.10)	**1.17 (1.06, 1.31)**	1.04 (0.98, 1.11)
Third trimester	−7.89 (−17.54, 1.75)	−0.01 (−0.04, 0.02)	0.02 (−0.03, 0.07)	1.01 (0.93,1.09)	**1.17 (1.05,1.31)**	1.05 (0.98,1.12)
Total pregnancy	−7.48(−17.48, 2.52)	−0.01 (−0.04, 0.02)	0.02 (−0.03, 0.07)	1.01 (0.93, 1.10)	**1.19 (1.06, 1.33)**	1.04 (0.98, 1.12)
**PM**_**10**_**_other (**μg**/m3)**						
First trimester	−3.76 (−15.46, 7.92)	0.03 (−0.01, 0.07)	**0.07 (0.01, 0.13)**	1.01 (0.92, 1.12)	1.15 (0.98, 1.34)	1.05 (0.97, 1.14)
Second trimester	−4.56 (−16.44, −7.31)	−0.02 (−0.06, 0.02)	0.01 (−0.04, 0.07)	1.03 (0.93,1.14)	**1.19 (1.02, 1.39)**	1.08 (1.00, 1.18)
Third trimester	−3.80 (−15.58, 7.99)	0.01 (−0.03, 0.04)	0.03 (−0.03, 0.09)	1.04 (0.94,1.15)	**1.23 (1.05, 1.44)**	**1.14 (1.05, 1.24)**
Total pregnancy	−8.02 (−20.03, 4.00)	0.01 (−0.03, 0.04)	0.04 (−0.02, 0.10)	1.04 (0.94, 1.15)	**1.20 (1.03, 1.41)**	**1.10 (1.01, 1.19)**
**PM**_**10**_**_total (μg/m3)**						
First trimester	2.73 (−11.41, 16.87)	**0.09 (0.05, 0.14)**	**0.16 (0.09, 0.23)**	0.98 (0.87,1.11)	0.98 (0.80, 1.20)	0.94 (0.85, 1.04)
Second trimester	−7.03 (−21.23, 7.17)	**−0.06 (−0.10, −0.01)**	0.00 (−0.07, 0.08)	0.96 (0.85,1.09)	1.12 (0.92,1.36)	1.02 (0.92, 1.13)
Third trimester	−7.81 (−19.9, 4.29)	0.00 (−0.03, 0.04)	0.02 (−0.03, 0.08)	1.06 (0.96, 1.18)	**1.24 (1.05, 1.47)**	**1.15 (1.06, 1.25)**
Total pregnancy	**−16.05 (−28.21, −3.88)**	0.01 (−0.03,0.05)	**0.09 (0.03, 0.15)**	1.10 (0.99, 1.22)	1.19 (1.00, 1.42)	1.07 (0.98,1.17)

Model adjusted for infant’s sex, maternal age, pre-pregnancy maternal body mass index (BMI), maternal educational level, maternal smoking during first 3 months of pregnancy, and environmental tobacco smoke exposure during pregnancy.

^***a***^represents the increase in odds of PTB for a per IQR increase in PM_10_ exposure;

^***b***^represents the increase in odds of TLBW for a per IQR increase in PM_10_ exposure;

^***c***^represents the increase in odds of SGA for a per IQR increase in PM_10_ exposure

The small associations in T1 between HC and PM_10__total, and BL for PM_10__other and PM_10__total remained similar after imputation (See Table 4 for full details).

## Discussion

We analysed associations between source-specific PM_10_ in pregnancy and birth outcomes in up to 12,000 singleton children born in south-west England in the early 1990s, with a full maternal residential history during pregnancy. We found increased odds for TLBW of 17–19% per IQR in relation to road traffic PM_10_ in each trimester. We saw similar associations with TLBW for PM_10_ from other local sources after multiple imputation for missing confounders. Both PM_10__road and PM_10__other exposures were highly correlated between trimesters, making it harder to determine a susceptible trimester. Maternal exposure to total PM_10_ (which was not highly correlated between trimesters) was also associated with increased risk of both TLBW and SGA, but this was driven by exposures in the third trimester. We additionally found some evidence for very small increases in HC and BL with higher PM_10_ exposures.

### Comparison of results with other studies

Results specific to T3 are biologically plausible since the third trimester is the period of greatest fetal weight gain [35–37] supported from the previous meta-analysis [12, 38]. Also, our findings are supported by the biological mechanism hypothesized for maternal exposure to cigarette smoking during pregnancy [39–41]. However, the biological mechanisms by which PM air pollution could affect fetal growth are not yet clearly understood. It is proposed that maternal exposure to PM_10_ in later stage of pregnancy is associated with maternal cardiovascular alterations in blood viscosity and coagulability [42, 43]. This may lead to a reduced utero-placental blood flow, thus impairing oxygen and nutrition transfer and consequently resulting in restricted fetal growth [44, 45].

Our finding of negative effects of total PM_10_ in T3 on TLBW in the 1990s are larger per unit mass PM_10_ than in a large study in London for 2006–10 based on birth registration data, which had lower concentrations of PM_10_ (mean 23.1 μg/m^3^ in London for WP, mean 32.6 μg/m^3^ in this study in Bristol in the early 1990s) [23]. Converting effect estimates to the same PM_10_ units, Smith et al. found odds of TLBW increased 4% (95% CI: 0.98, 1.11) per 10 μg/m^3^ increase in T3 PM_10_ exposure (See Supplementary Table 10 in Smith et al.), while we found the odds of TLBW increased 67% (95% CI: 1.19, 2.35) per 10 μg/m^3^ increase in T3 PM_10_ exposure. We found higher ORs than in the ESCAPE study for TLBW and total PM_10_ exposure in WP but the confidence intervals overlapped (ESCAPE study OR: 1.16, 95% CI 1.00, 1.35; our study OR: 1.32, 95% CI: 0.71, 2.43 per 10 μg/m^3^ increase in PM_10_); unlike our study, the ESCAPE analyses found little evidence of increased ORs for TLBW for T3 exposures [46].

Compared with the ESCAPE and London studies [23, 46], our study had reduced exposure misclassification through use of a full residential history and was based on daily modelled exposure values (as compared with assigned exposure to residence at birth and average monthly concentrations of air pollution), and improved confounder control with individual-level adjustment for pre-pregnancy BMI, maternal education level and environmental tobacco smoke exposure during pregnancy – this is likely to lead to higher estimates of effect size. However, there remains the possibility of residual confounding, including from dietary changes over time, which may potentially lead to an under- or over-estimate of the effects of PM_10_ exposure on birth weight.

To date, few studies have explored the associations between modelled source-specific PM exposures and adverse birth weight outcomes. Road traffic has been indicated as a potential source for adverse birth outcomes [23, 47]. Smith et al., reported that the association with PM_2.5_ traffic exhaust on TLBW was 2% stronger than that of PM_2.5_ traffic non-exhaust (See Supplementary Table 4 in Smith et al.) [23]. A Norwegian study applied a dispersion model [47] to women living in Oslo giving birth during 1999–2002 and found no clear associations between traffic pollution and TLBW. After multiple imputation, we also found evidence for associations with non-road local sources of PM_10_ exposures on TLBW.

An interaction between air pollution and child’s sex on birth weight was not found in this analysis but has been reported in other studies [48, 49]. Previous studies have reported boys are more likely to have lower birth weight in relation to prenatal exposure to air pollution [48, 49]. A systematic review summarized 11 studies and hypothesized that male fetuses may be susceptible to maternal PM exposure in pregnancy, with increased blood viscosity causing placental dysfunction [50]. We did not find clear evidence for interactions with social class as measured by mother’s educational level. Children of women classified to have low educational attainment had children with lower birthweight for PM_10__other but no associations with TLBW or with other PM measures. They also had reduction of BL for with PM_10__road but no interactions were seen with other outcomes. These may therefore have been chance findings. This is consistent with studies such as the ESCAPE study [46], which did not find clear interactions with educational level.

HC at birth is an important measurement because of the potential effects of air pollution on neurodevelopment [51]. Few previous studies have examined the relation between trimester-specific PM exposure during pregnancy and HC. One cohort study in the Netherlands looking at births in 2001–05 examined the relation between air pollution exposures with fetal growth measured by ultrasound during pregnancy and found higher levels of PM_10_ exposure in T3 were associated with reduction in fetal HC (− 0.18 mm, 95% CI: - 0.24, − 0.12 mm for per 1.0 μg/m^3^ increase in PM_10_) [24]. Another study in Australia did not detect associations between exposure to PM_10_ or other pollutants with HC and weight at birth [52]. We observed unexpected, albeit very small, apparent positive associations of PM_10_ air pollution with HC and BL. These are of unclear clinical importance and may be chance effects and are somewhat contradicted by the simultaneous finding of an increased risk of TLBW. We did not examine fetal loss in these analyses, but a possible explanation for this is that the most exposed fetuses who are more vulnerable might have been lost if exposed to higher air pollution exposure in early pregnancy.

### Strengths and limitations

A major strength of this study is that we had a full reconstructed residential history, accounting for residential mobility for each study cohort member using a cohort contact database [53] and were able to use this to assign PM_10_ exposure. We note that a recent paper from Scotland by Clemens et al. looking at births between 2002 and 11, did not find associations between birth weight and PM_10_ [54]. The authors used modelled PM_10_ concentration estimates based on calendar year average at a spatial resolution of 1 km × 1 km for PM_10_ and assigned PM_10_ based on postcode of address at the time of birth, which may have introduced significant misclassification of exposure and potentially explain failure to detect associations with birth weight. However, we acknowledge that in our study, errors, gaps, and overlaps of address history may still result in some exposure misclassification. Further, as information on participant’s time-activity pattern was not available, exposure estimates in this study refer only to ambient concentrations at home addresses while other exposure concentrations of each activity space were not considered (i.e. indoor, workplace or commuting).

A major limitation of this study is the missing data on some key confounding variables. We reported percentage of missing confounder variables before imputation (Table 1) which indicate that adjustments for these confounders dramatically reduced the study population and effect size in the fully adjusted models. To examine selection bias, we compared the characteristics for those births with missing and non-missing birth weight data (eTable 8 in the Supplement). We did not observe significant differences birth weight between groups.

To examine impact of missing confounders we conducted imputation analyses addressing this. Results from imputation analysis strengthened our main findings including larger effect sizes seen with birth weight, TLBW and SGA, with narrower confidence interval. Also, for all imputed models, we found a reasonable low fraction of missing information (FMI) < 0.53 (eTable 9 in the Supplement) which indicates low variability between imputed data sets and points at much of the “missing” information being captured by more completely observed variables [34].

Another potential limitation is that we pre-defined exposure windows of clinically defined trimesters. However, sensitive periods may be shorter or longer than 3 months or to exist in the overlap of multiple trimesters. It is suggested that a distributed lag model (DLM) [55, 56], may provide unbiased estimates and added flexibility to identify relevant time windows [57]. We only had access to the aggregated trimester-specific exposure data and therefore did not have enough exposure time windows to be able to run these models.

For PTBs, the third trimester is of very different lengths compared with term infants. To ensure equal length of exposures, we restricted whole-pregnancy exposures to 37 weeks to ensure equal length for TLBW and SGA, the results remained consistent (eTable 5 in the Supplement).

Air quality has improved in the UK over the last decades and there have been some change in sources, with increasing traffic contributions. However, our findings are likely to remain relevant to countries at similar stages of development. They also provide information on the ALSPAC cohort with very long-term active follow-up that is readily accessible to researchers, so findings could be useful in future life course analyses of environmental impacts on health from pre-birth onwards.

Although able to model source-specific PM_10_, a further limitation is that we were unable to model PM_2.5_ as PM_2.5_ monitoring was very limited in the UK until the 2000s, with no PM_2.5_ measurements in the study area until 2008.

Finally, this study assumed the date of conception is 2 weeks after the self-reported last menstrual period (LMP) approximate date. Additionally, exposure during periods preceding the conception was not considered. This may lead to an over- or under-estimate of the length of gestation, an inaccurate exposure estimate in each trimester and subsequently it may result in effects of each trimester being larger than effects of the entire pregnancy period averaged [58].

## Conclusion

This study supports a differential effect of maternal PM_10_ exposures on birth weight outcomes, with particular concern about exposures to PM_10_ from road transport sources and results for total PM_10_ point to greatest effect in the third trimester. Mitigation and prevention policies in ambient air pollution management are important to reduce the burden of TLBW and PTB, which have potential for lifelong impacts on morbidity and future mortality risks at the population level.

## Supplementary Information

**Additional file 1.**

## Data Availability

The ALSPAC dataset is available to all researchers on application to ALSPAC data and samples. Please note that the study website contains details of all the data that is available through a fully searchable data dictionary and variable search tool” and reference the following webpage: http://www.bristol.ac.uk/alspac/researchers/our-data/

## Abbreviations

ALSPAC: Avon Longitudinal Study of Parents and Children
BL: Birth length
CI: Confidence interval
CVDs: Cardiovascular diseases
FMI: Fraction of missing information
HC: Head circumference
MCMC: Markov chain Monte Carlo.
MI: Multiple imputation
MVN: Multivariate normal
OR: Odds ratio
PM: Particulate matter
PTB: Preterm birth
SGA: Small for gestational age
TLBW: Term low birth weight
T1: First trimester
T2: Second trimester
T3: Third trimester
WP: Whole pregnancy
