# Supplementary material for: Trimester effects of source-specific PM10 on birth weight outcomes in the Avon Longitudinal Study of Parents and Children (ALSPAC)
Source: Environ Health. 2021 Jan 7;20:4. doi: 10.1186/s12940-020-00684-w (PMC7788701; doi:10.1186/s12940-020-00684-w)
Supplement: Supplementary file 1 — Additional file 1. [file 12940_2020_684_MOESM1_ESM.zip › Alspac-BW-Envir health - supplement- 2nd Revision 27 NovR2.docx]

**Trimester effects of source-specific PM_10_ on birthweight outcomes in the Avon Longitudinal Study of Parents and Children (ALSPAC)**

Yingxin Chen^1^, Susan Hodgson^2^, John Gulliver^1, 2^, Raquel Granell^3^, A John Henderson^3^, Yutong Cai^4,5^, Anna L Hansell^1, 2^

1: Centre for Environmental Health and Sustainability, University of Leicester, Leicester, UK

2: MRC-PHE Centre for Environment and Health, Department of Epidemiology and Biostatistics, School of Public Health, Imperial College London, London, UK

3: MRC Integrative Epidemiology Unit, Population Health Sciences, Bristol Medical School, University of Bristol, Bristol, UK

4: The George Institute for Global Health, University of Oxford, Oxford, UK

5: Deep Medicine Programme, Oxford Martin School, University of Oxford, Oxford, UK

# Supplemental Material

## eTable 1. Distributions of PM_10_ exposure levels in each trimester and combined across pregnancy from difference sources based on home address (N=12, 020)

| PM_10_ exposure level (µg/m^3^) | Minimum | 25^th^ percentile | Mean ± SD | Median ± IQR | 75^th^ percentile | Maximum |
| --- | --- | --- | --- | --- | --- | --- |
| Local traffic source | | | | | | |
| First trimester | 0.07 | 0.48 | 0.96±0.71 | 0.80±0.73 | 1.20 | 7.80 |
| Second trimester | 0.10 | 0.48 | 0.96±0.71 | 0.80±0.71 | 1.19 | 8.37 |
| Third trimester | 0.06 | 0.46 | 0.94±0.69 | 0.78±0.70 | 1.17 | 7.26 |
| Total pregnancy | 0.13 | 0.46 | 0.95±0.69 | 0.81±0.72 | 1.19 | 7.69 |
| local industry/long-range particulates | | | | | | |
| First trimester | 0.78 | 3.87 | 5.24±1.97 | 5.10±2.40 | 6.29 | 18.36 |
| Second trimester | 0.79 | 3.88 | 5.25±1.96 | 5.08±2.41 | 6.29 | 19.07 |
| Third trimester | 0.86 | 3.81 | 5.16±1.94 | 5.05±2.41 | 6.23 | 19.32 |
| Total pregnancy | 1.12 | 3.88 | 5.22±1.85 | 5.17±2.29 | 6.17 | 17.09 |
| Total source | | | | | | |
| First trimester | 20.38 | 29.44 | 33.46±5.32 | 32.93±7.92 | 37.36 | 54.21 |
| Second trimester | 20.65 | 28.89 | 32.95±5.41 | 31.90±8.07 | 36.96 | 56.78 |
| Third trimester | 17.08 | 27.64 | 31.28±5.12 | 30.25±6.54 | 34.18 | 67.84 |
| Total pregnancy | 22.83 | 30.68 | 32.56±2.97 | 32.60±3.79 | 34.47 | 48.42 |

## eTable 2 Pearson’s correlations of PM10_road and PM10_other for same pregnancy period (T1, T2, T3 and WP)

|  | T1 | T2 | T3 | WP |
| --- | --- | --- | --- | --- |
|  | **PM_10__road** | | | |
| PM_10__other | 0.5489 | 0.5503 | 0.5531 | 0.5433 |

## eTable 3a Associations between source-specific PM_10_ exposure in different pregnancy periods and adverse birth outcomes (Models adjusted for interaction with maternal education)

| Per IQR increase | Birth weight, grams | Interaction | HC, cm | Interaction | BL, cm | Interaction |
| --- | --- | --- | --- | --- | --- | --- |
| in exposure | (N=7,683) |  | (N=6,127) |  | (N=5,037) |  |
| **PM_10__road (µg/m^3^)** | |  |  |  |  |  |
| First trimester | -1.85 (-16.27, 12.56) | 16.09 (-8.75,40.93) | 0.01 (-0.04, 0.05) | 0.01 (-0.07, 0.09) | 0.00 (-0.07, 0.07) | **0.13 (0.00, 0.25)** |
| Second trimester | -5.72 (-19.82, 8.38) | 20.07 (-4.22, 44.36) | -0.02 (-0.07, 0.02) | 0.02 (-0.06, 0.09)  0a | -0.03 (-0.10, 0.04) | **0.15 (0.03, 0.27)** |
| Third trimester | -5.03 (-19.29, 9.24) | 20.09 (-4.025, 44.42) | -0.01 (-0.06, 0.03) | 0.03 (-0.04, 0.11) | -0.01 (-0.08, 0.06) | **0.14 (0.02, 0.26)** |
| Total pregnancy | -4.78 (-19.58, 10.03) | 20.24 (-5.18, 45.65) | -0.01 (0.06, 0.04) | 0.02 (-0.06, 0.10) | -0.02 (-0.09, 0.06) | **0.15 (0.02, 0.28)** |
| **PM_10__other (µg/m^3^)** | |  |  |  |  |  |
| First trimester | 6.47 (-11.84, 24.77) | 16.66 (-11.15, 44.47) | 0.04 (-0.02, 0.10) | 0.03 (-0.06, 0.12) | 0.09 (-0.01, 0.18) | 0.03 (-0.11, 0.17) |
| Second trimester | -1.83 (-20.11, 16.45) | 26.04 (-2.02, 54.09) | -0.03 (-0.09, 0.03) | 0.08 (-0.01, 0.16) | 0.01 (-0.09, 0.10) | 0.09 (-0.05, 0.23) |
| Third trimester | -6.98 (-25.65, 11.69) | **38.05 (9.40, 66.70)** | -0.01 (-0.07, 0.05) | 0.09 (0.00, 0.18) | 0.02 (-0.08, 0.11) | 0.12 (-0.03, 0.26) |
| Total pregnancy | -1.27 (19.86, 17.32) | **28.59 (0.32, 56.86)** | 0.00 (-0.06, 0.06) | 0.07 (-0.02, 0.16) | 0.04 (0.05, 0.14) | 0.08 (-0.06, 0.23) |
| **PM_10__total (µg/m^3^)** | |  |  |  |  |  |
| First trimester | 5.20 (-15.75, 26.14) | 10.02 (-24.71, 44.76) | **0.09 (0.02, 0.15)** | 0.03 (-0.08, 0.13) | 0.15 (0.04, 0.25) | 0.00 (-0.17, 0.17) |
| Second trimester | 0.28 (-20.78, 21.35) | 14.33 (-20.38, 49.03) | **-0.07 (-0.14, 0.00)** | 0.10 (-0.01, 0.21) | 0.00 (-0.11, 0.12) | 0.08 (-0.11, 0.26) |
| Third trimester | -7.32 (25.52, 10.88) | 22.96 (-7.12, 53.03) | 0.00 (-0.06, 0.05) | 0.03 (-0.06, 0.12) | 0.02 (-0.07, 0.10) | 0.01 (-0.13, 0.16) |
| Total pregnancy | -9.60 (-27.99, 8.79) | 27.38 (-2.31, 57.06) | -0.01 (-0.06, 0.05) | 0.09 (-0.01, 0.18) | 0.08 (-0.01, 0.18) | 0.05 (-0.10, 0.20) |
| ***^a^***represents the increase in odds of PTB for a per IQR increase in PM_10_ exposure; | | | | | | |
| ***^b^***represents the increase in odds of TLBW for a per IQR increase in PM_10_ exposure; | | | | | | |
| ***^c^***represents the increase in odds of SGA for a per IQR increase in PM_10_ exposure | | | | | | |
| Model adjusted for infant’s sex, maternal age, pre-pregnancy maternal body mass index (BMI), maternal educational level, maternal smoking during first 3 months of pregnancy, and environmental tobacco smoke exposure during pregnancy, interaction with maternal education | | | | | | |

## eTable 3a (Continued) Associations between source-specific PM_10_ exposure in different pregnancy periods and adverse birth outcomes (Models adjusted for interaction with maternal education)

| Per IQR increase | PTB*^a^* (case: 601) | Interaction | TLBW*^b^* (case: 271) | Interaction | SGA***^c^*** (case: 928) | Interaction |
| --- | --- | --- | --- | --- | --- | --- |
| in exposure | (N=7,761) |  | (N=7,350) |  | (N=7,683) |  |
| **PM_10__road (µg/m^3^)** | |  |  |  |  |  |
| First trimester | 1.02 (0.89, 1.16) | 0.92 (0.71, 1.19) | **1.19 (1.01, 1.41)** | 0.83 (0.55, 1.24) | 1.05 (0.95, 1.16) | 0.88 (0.71, 1.08) |
| Second trimester | 1.03 (0.91, 1.18) | 0.88 (0.68, 1.14) | **1.20 (1.02, 1.42)** | 0.91 (0.63, 1.31) | 1.07 (0.97, 1.18) | 0.87 (0.71, 1.06) |
| Third trimester | 1.01 (0.88,1.15) | 0.93 (0.72, 1.20) | **1.19 (1.01,1.42)** | 0.90 (0.62, 1.32) | 1.07 (0.97,1.18) | 0.89 (0.73, 1.09) |
| Total pregnancy | 1.02 (0.89, 1.18) | 0.91 (0.70, 1.18) | **1.21 (1.02, 1.44)** | 0.88 (0.59, 1.30) | 1.07 (0.97, 1.19) | 0.87 (0.70, 1.08) |
| **PM_10__other (µg/m^3^)** | |  |  |  |  |  |
| First trimester | 0.99 (0.84, 1.19) | 0.94 (0.71, 1.25) | 1.12 (0.86, 1.45) | 0.73 (0.44, 1.20) | 1.01 (0.88, 1.15) | 0.98 (0.78, 1.23) |
| Second trimester | 1.05 (0.88, 1.24) | 0.85 (0.64, 1.13) | 1.23 (0.96, 1.57) | 0.84 (0.52, 1.34) | 1.05 (0.92, 1.20) | 1.00 (0.80, 1.26) |
| Third trimester | 1.06 (0.89,1.26) | 0.94 (0.70, 1.15) | 1.28 (0.99, 1.66) | 0.81 (0.50, 1.31) | 1.10 (0.96, 1.26) | 0.97 (0.77, 1.22) |
| Total pregnancy | 1.04 (0.87, 1.23) | 0.90 (0.68, 1.20) | 1.22 (0.94, 1.57) | 0.78 (0.48, 1.27) | 1.06 (0.92, 1.21) | 0.98 (0.78, 1.23) |
| **PM_10__total (µg/m^3^)** | |  |  |  |  |  |
| First trimester | 1.01 (0.83,1.23) | 0.88 (0.62, 1.25) | 0.88 (0.64, 1.20) | 0.59 (0.30, 1.14) | 0.94 (0.80, 1.10) | 0.88 (0.65, 1.18) |
| Second trimester | 0.90 (0.74,1.10) | 0.80 (0.55, 1.15) | 1.17 (0.86,1.59) | 0.73 (0.39, 1.36) | 1.02 (0.87, 1.19) | 0.95 (0.71, 1.27) |
| Third trimester | 1.05 (0.88, 1.24) | 1.09 (0.81, 1.47) | **1.46 (1.13, 1.89)** | 0.84 (0.51, 1.38) | **1.16 (1.01, 1.32)** | 1.06 (0.83, 1.34) |
| Total pregnancy | 1.06 (0.88, 1.26) | 0.86 (0.64, 1.17) | 1.27 (0.97, 1.67) | 0.61 (0.36, 1.02) | 1.07 (0.93,1.22) | 0.94 (0.73, 1.20) |
| ***^a^***represents the increase in odds of PTB for a per IQR increase in PM_10_ exposure; | | | | | | |
| ***^b^***represents the increase in odds of TLBW for a per IQR increase in PM_10_ exposure; | | | | | | |
| ***^c^***represents the increase in odds of SGA for a per IQR increase in PM_10_ exposure | | | | | | |
| Model adjusted for infant’s sex, maternal age, pre-pregnancy maternal body mass index (BMI), maternal educational level, maternal smoking during first 3 months of pregnancy, and environmental tobacco smoke exposure during pregnancy, interaction with maternal education | | | | | | |

## eTable 3b Association of PM_10_ exposures with birth outcomes stratified by maternal education for outcomes with significant exposure*maternal education interaction term in table 3a

| Per IQR increase in exposure | Birth weight, grams (N=7,683) | |
| --- | --- | --- |
|  | Maternal education: O level or below | Maternal education: A level or above |
| **PM_10__other (µg/m^3^)** | |  |
| Third trimester | -6.52 (-25.65, 12.61) | **28.84 (7.91, 49.76)** |
| Total pregnancy | -0.74 (-19.78, 4.09) | **25.14 (4.62, 45.65)** |
| Model adjusted for infant’s sex, maternal age, pre-pregnancy maternal body mass index (BMI), maternal smoking during first 3 months of pregnancy, and environmental tobacco smoke exposure during pregnancy, stratified by maternal education. | | |

| Per IQR increase in exposure | BL, cm (N=5,037) | |
| --- | --- | --- |
|  | Maternal education: O level or below | Maternal education: A level or above |
| **PM_10__road (µg/m^3^)** | |  |
| First trimester | 0.00 (-0.07, 0.07) | **0.12 (0.02, 0.22)** |
| Second trimester | -0.03 (-0.10, 0.04) | **0.11 (0.01, 0.20)** |
| Third trimester | -0.01 (-0.08, 0.06) | **0.13 (0.03, 0.22)** |
| Total pregnancy | -0.01 (-0.09, 0.06) | **0.12 (0.03, 0.23)** |
| Model adjusted for infant’s sex, maternal age, pre-pregnancy maternal body mass index (BMI), maternal smoking during first 3 months of pregnancy, and environmental tobacco smoke exposure during pregnancy, stratified by maternal education | | |

## eTable 4. The adjusted associations between PM_10__total trimester average exposures (TAEs) and adverse birth outcomes

|  | Mean difference | | | Odd ratios | | |
| --- | --- | --- | --- | --- | --- | --- |
| PM_10_ exposure | Birth weight, grams (95% CI) (N=7,683) | Head circumference, cm (95% CI) (N=6,309) | Birth length, cm (95% CI) (N=5,185) | PTB***^a^*** (95% CI) (N=7,984) | TLBW***^b^*** (95% CI) (N=7,350) | SGA***^c^*** (95% CI) (N=7,493) |
| PM_10__total |  |  |  |  |  |  |
| T1 | 9.18 (-7.62, 25.97) | **0.10 (0.04, 0.15)** | **0.15 (0.07, 0.23)** | 0.97 (0.83, 1.14) | 0.80 (0.61, 1.07) | 0.92 (0.81, 1.05) |
| T2 | 5.77 (-11.00,22.53) | -0.03 (-0.08, 0.02) | 0.04 (-0.05, 0.13) | **0.83 (0.70, 0.98)** | 1.07 (0.81, 1.43) | 0.99 (0.87, 1.14) |
| T3 | 1.64 (-12.92, 16.21) | 0.02 (-0.03, 0.06) | 0.03 (-0.04, 0.10) | 1.08 (0.94, 1.25) | **1.35 (1.08, 1.68)** | **1.17 (1.04, 1.30)** |
| Note: Results are taken from mutually adjusted models, in which exposure variables PM_10__total TAEs were simultaneously entered in the regression to evaluate their independent effects on adverse birth outcomes, with additional adjustment for gestational weeks (only for birth weight), infant's sex, and maternal age, pre-pregnancy maternal BMI, maternal educational level, maternal smoke during first 3 months of pregnancy and environmental tobacco smoke exposure during pregnancy  ***^a^***represents the increase in odds of PTB for a per IQR increase in PM_10__total;  ***^b^***represents the increase in odds of TLBW for a per IQR increase in PM_10__total;  ***^c^***represents the increase in odds of SGA for a per IQR increase in PM_10__total. | | | | | | |

## eTable 5 The adjusted associations between PM_10__road and PM_10__other for same pregnancy period (T1, T2, T3 and WP)

|  | Mean difference | | | Odd ratios | | |
| --- | --- | --- | --- | --- | --- | --- |
| PM_10_ exposure | Birth weight, grams (95% CI) (N=7,683) | Head circumference, cm (95% CI) (N=6,309) | Birth length, cm (95% CI) (N=5,185) | PTB***^a^*** (95% CI) (N=7,984) | TLBW***^b^*** (95% CI) (N=7,350) | SGA***^c^*** (95% CI) (N=7,493) |
| First trimester |  |  |  |  |  |  |
| Road | -3.89 (-17.87, 10.09) | -0.02 (-0.06, 0.03) | 0.00 (-0.07, 0.07) | 1.01 (0.89, 1.16) | **1.19 (1.00, 1.42)** | 1.03 (0.93, 1.14) |
| Other | 16.14 (-0.28, 32.56) | **0.06 (0.02, 0.11)** | **0.10 (0.02, 0.18)** | 0.96 (0.82, 1.13) | 0.90 (0.69, 1.17) | 0.98 (0.86, 1.12) |
| Second trimester |  |  |  |  |  |  |
| Road | -4.57 (-18.36, 9.22) | -0.02 (-0.07, 0.02) | 0.00 (-0.07, 0.06) | 1.00 (0.88, 1.14) | 1.16 (0.97, 1.38) | 1.02 (0.92, 1.13) |
| Other | 12.24 (-4.42, 28.90) | 0.02 (-0.03, 0.07) | 0.05 (-0.03, 0.13) | 0.98 (0.84, 1.16) | 1.05 (0.81, 1.35) | 1.03 (0.91, 1.18) |
| Third trimester |  |  |  |  |  |  |
| Road | -3.18 (-17.03, 10.66) | -0.02 (-0.06, 0.02) | 0.01 (-0.05, 0.08) | 0.96 (0.83, 1.10) | 1.12 (0.94, 1.35) | 1.00 (0.91, 1.12) |
| Other | 11.27 (-5.70, 28.25) | 0.04 (-0.01, 0.09) | 0.06 (-0.03, 0.14) | 1.06 (0.90, 1.25) | 1.11 (0.86, 1.44) | 1.08 (0.95, 1.22) |
| Whole Pregnancy |  |  |  |  |  |  |
| Road | -4.30 (-18.61, 10.01) | -0.02 (-0.07, 0.02) | 0.00 (-0.07, 0.07) | 0.99 (0.87, 1.14) | 1.17 (0.97, 1.40) | 1.02 (0.92, 1.13) |
| Other | 13.75 (-2.91, 30.40) | 0.04 (-0.01, 0.09) | 0.08 (-0.01, 0.16) | 1.00 (0.85, 1.18) | 1.02 (0.78, 1.32) | 1.03 (0.91, 1.18) |

## eTable 6. Associations between source-specific PM_10_ exposure in different pregnancy periods and adverse birth outcomes restricting study samples to all women whose gestation finished at 37 weeks

|  |  | | | |
| --- | --- | --- | --- | --- |
| Per IQR increase | Term low birthweight | | Small for gestational age | |
| in exposure | Unadjusted (N=11,302) | Adjusted (N=7,350) | Unadjusted (N=11,302) | Adjusted (N=7,171) |
| **PM_10__road (µg/m^3^)** | | | | |
| First trimester | **1.20 (1.06, 1.30)** | **1.15 (0.99, 1.34)** | 1.05 (0.98, 1.13) | 1.03 (0.94, 1.13) |
| Second trimester | **1.18 (1.06, 1.32)** | **1.18 (1.02, 1.37)** | 1.06 (0.99, 1.13) | 1.05 (0.96, 1.13) |
| Third trimester | **1.18 (1.06, 1.32)** | **1.17 (1.00, 1.36)** | **1.07 (1.01, 1.15)** | 1.06 (0.97, 1.15) |
| Total pregnancy | **1.20 (1.07, 1.34)** | **1.22 (1.00, 1.38)** | 1.07 (1.00, 1.14) | 1.05 (0.96, 1.15) |
| **PM_10__other (µg/m^3^)** |  |  |  |  |
| First trimester | 1.15 (0.99, 1.34) | 1.02 (0.82, 1.28) | 1.08 (1.00, 1.17) | 1.02 (1.00, 1.14) |
| Second trimester | **1.19 (1.02, 1.39)** | 1.16 (0.94, 1.44) | **1.10 (1.02, 1.20)** | 1.07 (0.96, 1.19) |
| Third trimester | **1.23 (1.05, 1.43)** | 1.20 (0.97, 1.49) | **1.18 (1.08, 1.27)** | 1.11 (1.00, 1.24) |
| Total pregnancy | **1.20 (1.03, 1.40)** | 1.13 (0.91, 1.41) | **1.13 (1.04, 1.22)** | 1.07 (0.96, 1.20) |
| **PM_10__total (µg/m^3^)** |  |  |  |  |
| First trimester | 0.99 (0.82, 1.22) | 0.77 (0.58, 1.01) | 0.96 (0.87, 1.07) | 0.92 (0.80, 1.05) |
| Second trimester | 1.11 (0.91, 1.35) | 1.08 (0.83, 1.41) | 1.01 (0.91, 1.12) | 1.01 (0.88, 1.15) |
| Third trimester | **1.24 (1.05, 1.46)** | **1.39 (1.12, 1.74)** | **1.20 (1.10, 1.31)** | **1.21 (1.08, 1.36)** |
| Total pregnancy | **1.20 (1.01, 1.42)** | **1.11 (0.88, 1.40)** | **1.09 (1.00, 1.20)** | 1.07 (0.96, 1.21) |
| Model adjusted for infant’s sex, maternal age, pre-pregnancy maternal body mass index (BMI), maternal educational level, maternal smoking during  first 3 months of pregnancy, and environmental tobacco smoke exposure during pregnancy | | | | |

## eTable 7 Associations between source-specific PM_10_ exposure in different pregnancy periods and adverse birth outcomes restricting study samples to first child

|  | | | | | | |
| --- | --- | --- | --- | --- | --- | --- |
|  | Mean difference | | | Odd ratios | | |
| Per IQR increase | Birth weight, grams | Head Circumference, cm | Birth length, cm | PTB***^a^***  Case: 601 | TLBW***^b^***  Case: 271 | SGA***^c^***  Case: 928 |
| in exposure | (N=2,949) | (N=2,450) | (N=2,087) | (N=2,982) | (N=2,785) | (N=2,949) |
| **PM_10__road (µg/m^3^)** | |  |  |  |  |  |
| First trimester | 5.47 (-13.56, 24.50) | 0.02 (-0.04, 0.08) | 0.02 (-0.07, 0.11) | 0.99 (0.84, 1.17) | 1.15 (0.92, 1.43) | 0.96 (0.84, 1.10) |
| Second trimester | 4.53 (-14.56, -23.62) | 0.00 (-0.06, 0.06) | 0.00 (-0.09, 0.09) | 0.99 (0.83, 1.17) | 1.18 (0.95, 1.47) | 1.01 (0.89, 1.15) |
| Third trimester | 6.29 (-12.74, 25.31) | 0.02 (-0.04, 0.08) | 0.04 (-0.06, 0.13) | 0.97 (0.82,1.14) | 1.11 (0.88,1.40) | 1.00 (0.88,1.13) |
| Total pregnancy | 5.65 (-14.22, 25.51) | 0.01 (-0.05, 0.07) | 0.02 (-0.07, 0.12) | 0.98 (0.83, 1.17) | 1.16 (0.92, 1.46) | 0.99 (0.87, 1.13) |
| **PM_10__other (µg/m^3^)** | |  |  |  |  |  |
| First trimester | **25.38 (3.70, 47.06)** | 0.07 (-0.01, 0.07) | **0.11 (0.01, 0.22)** | 0.89 (0.73, 1.08) | 0.95 (0.70, 1.29) | 0.90 (0.77, 1.05) |
| Second trimester | 18.91 (-3.41, 41.23) | 0.01 (-0.04, 0.10) | 0.05 (-0.06, 0.16) | 0.90 (0.74,1.10) | 1.04 (0.77, 1.40) | 1.00 (0.86, 1.17) |
| Third trimester | 21.17 (-1.30, 43.64) | 0.06 (-0.01, 0.13) | 0.10 (-0.01, 0.21) | 0.92 (0.75,1.12) | 1.06 (0.79, 1.43) | 0.98 (0.84, 1.15) |
| Total pregnancy | **22.97 (0.73, 45.22)** | 0.06 (-0.01, 0.13) | 0.10 (-0.01, 0.21) | 0.90 (0.73, 1.10) | 1.01 (0.75, 1.37) | 0.96 (0.82, 1.12) |
| **PM_10__total (µg/m^3^)** | |  |  |  |  |  |
| First trimester | 16.85 (-9.89, 43.58) | **0.13 (0.04, 0.21)** | **0.16 (0.03, 0.29)** | 0.91 (0.72,1.15) | 0.76 (0.52, 1.10) | **0.81 (0.67, 0.97)** |
| Second trimester | 10.08 (-18.03, 38.20) | -0.02 (-0.11, -0.07) | -0.04 (-0.18, 0.11) | 0.80 (0.63,1.03) | 0.90 (0.61,1.33) | 1.04 (0.86, 1.26) |
| Third trimester | -4.78 (-28.35, 18.79) | -0.02 (-0.09, 0.06) | 0.02 (-0.09, 0.13) | 1.10 (0.90, 1.34) | **1.36 (1.00, 1.84)** | **1.17 (1.00, 1.37)** |
| Total pregnancy | 5.44 (-17.91, 28.78) | 0.04 (-0.03,0.11) | 0.09 (0.03, 0.20) | 0.95 (0.78, 1.17) | 0.96 (0.70, 1.32) | 1.00 (0.85,1.17) |
| ***: p<0.01, **: p<0.05, *: p<0.1 | | | | | | |
| ***^a^***represents the increase in odds of Preterm birth for a per IQR increase in PM_10_ exposure; | | | | | | |
| ***^b^***represents the increase in odds of Term low birth weight for a per IQR increase in PM_10_ exposure; | | | | | | |
| ***^c^***represents the increase in odds of Small for gestational age for a per IQR increase in PM_10_ exposure | | | | | | |
| Model adjusted for infant’s sex, maternal age, pre-pregnancy maternal body mass index (BMI), maternal educational level, maternal smoking during first 3 months of pregnancy, and environmental tobacco smoke exposure during pregnancy | | | | | | |

## eTable 8. Comparison of major confounders and outcomes for those with missing and non-missing outcome data and non-missing confounder data

|  | **Excluded from analyses due to missing birthweight data**  **(n=124)** | **Included in analyses***  **(n = 11,896)** | **Birth weight (grams)**  **Mean ± SD** | **p-value for difference** |
| --- | --- | --- | --- | --- |
| Infant’s sex |  |  |  |  |
| Male, n (%) | 65 (52.42) | 6,150 (51.70) | 3,467.97 ± 561.73 | 0.873 |
| Female, n (%) | 59 (47.58) | 5,746 (48.30) | 3,359.16 ± 510.09 |  |
| Maternal age, mean ± SD | 27.39 ± 5.31 | 27.79 ± 4.85 | - | 0.38 |
| Education |  |  |  | 0.554 |
| O level or below, n (%) | 72 (67.92) | 6,914 (65.17) | 3414.43 ± 537.13 |  |
| A level or above, n (%) | 34 (32.08) | 3,695 (34.83) | 3464.37 ± 507.05 |  |
| Missing data | 1,305 |  |  |  |
| Maternal BMI |  |  |  | 0.752 |
| BMI<18.5 | 7 (6.86) | 503 (5.10) | 3197.76 ± 510.93 |  |
| 18.5≤BMI<25, n (%) | 77 (75.49) | 7,282 (73.88) | 3415.95 ± 513.41 |  |
| 25≤BMI<30, n (%) | 13 (12.75) | 1,524 (15.46) | 3513.33 ± 539.53 |  |
| BMI≥30, n (%) | 5 (4.90) | 547 (5.55) | 3593.64 ± 622.71 |  |
| Missing data | 2,062 |  |  |  |
| Maternal smoking |  |  |  | 0.715 |
| Non-smoker | 85 (73.91) | 8,453 (75.39) | 3466.28 ± 527.18 |  |
| Smoker | 30 (26.09) | 2,760 (24.61) | 3284.85 ± 549.80 |  |
| Missing data | 692 |  |  |  |
| Environmental smoking |  |  |  | 0.95 |
| Not exposed | 36 (39.13) | 3,480 (38.81) | 3491.95 ± 502.52 |  |
| Exposed | 56 (60.87) | 5,487 (61.19) | 3391.21 ± 526.42 |  |
| Missing data | 2,961 |  |  |  |
| *Analysis for birthweight | | | | |

## eTable 9. Model checking in multiple imputation: largest fraction of missing information (FMI)

|  | Birth weight, grams | Head Circumference, cm | Birth length, cm | PTB***^a^***  Case: 601 | TLBW***^b^***  Case: 271 | SGA***^c^***  Case: 928 |
| --- | --- | --- | --- | --- | --- | --- |
|  | (N=11,896) | (N=9,245) | (N=7,583) | (N=12,020) | (N=11,302) | (N=11,896) |
| **PM_10__road (µg/m^3^)** | |  |  |  |  |  |
| First trimester | 0.38 | 0.22 | 0.30 | 0.31 | 0.37 | 0.40 |
| Second trimester | 0.32 | 0.30 | 0.23 | 0.30 | 0.34 | 0.37 |
| Third trimester | 0.30 | 0.13 | 0.23 | 0.48 | 0.47 | 0.37 |
| Total pregnancy | 0.25 | 0.25 | 0.23 | 0.26 | 0.45 | 0.31 |
| **PM_10__other (µg/m^3^)** | |  |  |  |  |  |
| First trimester | 0.23 | 0.34 | 0.21 | 0.25 | 0.33 | 0.39 |
| Second trimester | 0.35 | 0.40 | 0.29 | 0.34 | 0.36 | 0.37 |
| Third trimester | 0.40 | 0.39 | 0.35 | 0.35 | 0.39 | 0.39 |
| Total pregnancy | 0.34 | 0.27 | 0.25 | 0.26 | 0.30 | 0.38 |
| **PM_10__total (µg/m^3^)** | |  |  |  |  |  |
| First trimester | 0.29 | 0.28 | 0.38 | 0.21 | 0.29 | 0.37 |
| Second trimester | 0.33 | 0.39 | 0.45 | 0.34 | 0.47 | 0.27 |
| Third trimester | 0.32 | 0.41 | 0.40 | 0.46 | 0.34 | 0.42 |
| Total pregnancy | 0.21 | 0.38 | 0.27 | 0.30 | 0.52 | 0.25 |
| ***^a^***represents the increase in odds of Preterm birth for a per IQR increase in PM_10_ exposure; | | | | | | |
| ***^b^***represents the increase in odds of Term low birth weight for a per IQR increase in PM_10_ exposure; | | | | | | |
| ***^c^***represents the increase in odds of Small for gestational age for a per IQR increase in PM_10_ exposure | | | | | | |
