# Supplementary material for: Trimester effects of source-specific PM10 on birth weight outcomes in the Avon Longitudinal Study of Parents and Children (ALSPAC)
Source: Environ Health. 2021 Jan 7;20:4. doi: 10.1186/s12940-020-00684-w (PMC7788701; doi:10.1186/s12940-020-00684-w)
Supplement: Supplementary file 1 — Additional file 1. [file 12940_2020_684_MOESM1_ESM.zip › Supplement - 2nd Revision 27 Nov - TCR2.pdf]

Trimester effects of source-specific PM_{10} on birthweight outcomes in the Avon Longitudinal Study of Parents and Children (ALSPAC)

Yingxin Chen[1], Susan Hodgson[2], John Gulliver[1, 2], Raquel Granell[3], A John Henderson[3], Yutong Cai[4,5], Anna L Hansell[1, 2]

1: Centre for Environmental Health and Sustainability, University of Leicester, Leicester, UK

2: MRC-PHE Centre for Environment and Health, Department of Epidemiology and Biostatistics, School of Public Health, Imperial College London, London, UK

3: MRC Integrative Epidemiology Unit, Population Health Sciences, Bristol Medical School, University of Bristol, Bristol, UK

4: The George Institute for Global Health, University of Oxford, Oxford, UK

5: Deep Medicine Programme, Oxford Martin School, University of Oxford, Oxford, UK.

Supplemental Material

eTable 1. Distributions of PM_{10} exposure levels in each trimester and combined across pregnancy from difference sources based on home address (N=12, 020)

PM_{10} exposure level ($\mu g/m^3$)	Minimum	25th percentile	Mean ± SD	Median ± IQR	75th percentile	Maximum
Local traffic source						
First trimester	0.07	0.48	0.96±0.71	0.80±0.73	1.20	7.80
Second trimester	0.10	0.48	0.96±0.71	0.80±0.71	1.19	8.37
Third trimester	0.06	0.46	0.94±0.69	0.78±0.70	1.17	7.26
Total pregnancy	0.13	0.46	0.95±0.69	0.81±0.72	1.19	7.69
local industry/long-range particulates						
First trimester	0.78	3.87	5.24±1.97	5.10±2.40	6.29	18.36
Second trimester	0.79	3.88	5.25±1.96	5.08±2.41	6.29	19.07
Third trimester	0.86	3.81	5.16±1.94	5.05±2.41	6.23	19.32
Total pregnancy	1.12	3.88	5.22±1.85	5.17±2.29	6.17	17.09
Total source						
First trimester	20.38	29.44	33.46±5.32	32.93±7.92	37.36	54.21
Second trimester	20.65	28.89	32.95±5.41	31.90±8.07	36.96	56.78
Third trimester	17.08	27.64	31.28±5.12	30.25±6.54	34.18	67.84
Total pregnancy	22.83	30.68	32.56±2.97	32.60±3.79	34.47	48.42

eTable 2 Pearson's correlations of PM10_road and PM10_other for same pregnancy period (T1, T2, T3 and WP)

	T1	T2	T3	WP
	PM_{10}_road			
**PM_{10}_other**	0.5489	0.5503	0.5531	0.5433

eTable 3a AssociationsInteraction coefficients between maternal educationsource-specific PM_{10} exposure in different pregnancy periods and PM_{10} exposures for adverse birth outcomes (Models adjusted for interaction with maternal education)

	Mean difference			Odd ratios		
Per IQR increase in exposure	Birth weight, grams (N=7,683)	InteractionHC, cm (N=6,127)	BLHC, cm (N=5,0376,127)	PTB[a] (case: 601)Interaction (N=7,761)	TLBW[b] (case: 271)BL, cm (N=7,3505,037)	SGA[c] (case: 928)Interaction (N=7,683)
**PM_{10}_road (μg/m³)**						
First trimester	-1.85 (-16.09 (-	0.01 (-0.07, 016.09 (-	0.13 (01 (-0.0004,	0.92 (01 (-0.71,	0.83 (00 (-0.55,	**0.8813 (0.71, 1.0800, 0.25)**
Second trimester	-5.72 (-19.82, 8.38)	20.07 (-4.22, 44.36)	-0.15 (02 (-	0.88 (02 (-0.68,	-0.91 (03 (-0.63,	**0.8715 (0.71, 1.0603, 0.27)**
Third trimester	-5.03 (-19.29, 9.24)	20.09 (-4.025, 44.42)	-0.14 (01 (-	0.93 (03 (-0.72,	-0.90 (01 (-0.62,	**0.8914 (0.73, 1.0902, 0.26)**
Total pregnancy	-4.78 (-19.58, 10.03)	20.24 (-5.18, 45.65)	-0.1501 (0.0206,	0.91 (02 (-0.70,	-0.88 (02 (-0.59,	**0.8715 (0.70, 1.08)02, 0.28)**
**PM_{10}_other (μg/m³)**						
First trimester	6.47 (-11.84, 24.77)	16.66 (-11.15, 44.47)	0.04 (-0.02, 0.10)	0.03 (-0.06, 0.12)	0.09 (-0.01, 0.18)	0.03 (-0.11, 0.17)
Second trimester	-1.83 (-20.11, 16.45)	26.04 (-2.02, 54.09)	-0.03 (-0.09, 0.03)	0.08 (-0.01, 0.16)	0.01 (-0.09, 0.10)	0.09 (-0.05, 0.23)
Third trimester	-6.98 (-25.65, 11.69)	**38.05 (9.40, 66.70)**	-0.01 (-0.07, 0.05)	0.09 (0.00, 0.18)	0.02 (-0.08, 0.11)	0.12 (-0.03, 0.26)
Total pregnancy	-1.27 (19.86, 17.32)	**28.59 (0.32, 56.86)**	0.00 (-0.06, 0.06)	0.07 (-0.02, 0.16)	0.04 (0.05, 0.14)	0.08 (-0.06, 0.23)
**PM_{10}_total (μg/m³)**						
First trimester	5.20 (-15.75, 26.14)	10.02 (-24.71, 44.76)	**0.09 (0.02, 0.15)**	0.03 (-0.08, 0.13)	0.15 (0.04, 0.25)	0.00 (-0.17, 0.17)
Second trimester	0.28 (-20.78, 21.35)	14.33 (-20.38, 49.03)	**-0.07 (-0.14, 0.00)**	0.10 (-0.01, 0.21)	0.00 (-0.11, 0.12)	0.08 (-0.11, 0.26)
Third trimester	-7.32 (25.52, 10.88)	22.96 (-7.12, 53.03)	0.00 (-0.06, 0.05)	0.03 (-0.06, 0.12)	0.02 (-0.07, 0.10)	0.01 (-0.13, 0.16)
Total pregnancy	-9.60 (-27.99, 8.79)	27.38 (-2.31, 57.06)	-0.01 (-0.06, 0.05)	0.09 (-0.01, 0.18)	0.08 (-0.01, 0.18)	0.05 (-0.10, 0.20)

[a]represents the increase in odds of PTB for a per IQR increase in PM_{10} exposure;
[b]represents the increase in odds of TLBW for a per IQR increase in PM_{10} exposure;
[c]represents the increase in odds of SGA for a per IQR increase in PM_{10} exposure
Model adjusted for infant's sex, maternal age, pre-pregnancy maternal body mass index (BMI), maternal educational level, maternal smoking during first 3 months of pregnancy, and environmental tobacco smoke exposure during pregnancy, interaction with maternal education

eTable 3a (Continued) Associations between source-specific PM_{10} exposure in different pregnancy periods and adverse birth outcomes (Models adjusted for interaction with maternal education)

Per IQR increase in exposure	PTB[a] (case: 601) (N=7,761)	Interaction	TLBW[b] (case: 271) (N=7,350)	Interaction	SGA[c] (case: 928) (N=7,683)	Interaction
**PM_{10}_road ($\mu g/m^3$)**						
First trimester	1.02 (0.89, 1.16)	0.92 (0.71, 1.19)	**1.19 (1.01, 1.41)**	0.83 (0.55, 1.24)	1.05 (0.95, 1.16)	0.88 (0.71, 1.08)
Second trimester	1.03 (0.91, 1.18)	0.88 (0.68, 1.14)	**1.20 (1.02, 1.42)**	0.91 (0.63, 1.31)	1.07 (0.97, 1.18)	0.87 (0.71, 1.06)
Third trimester	1.01 (0.88,1.15)	0.93 (0.72, 1.20)	**1.19 (1.01,1.42)**	0.90 (0.62, 1.32)	1.07 (0.97,1.18)	0.89 (0.73, 1.09)
Total pregnancy	1.02 (0.89, 1.18)	0.91 (0.70, 1.18)	**1.21 (1.02, 1.44)**	0.88 (0.59, 1.30)	1.07 (0.97, 1.19)	0.87 (0.70, 1.08)
**PM_{10}_other ($\mu g/m^3$)**						
First trimester	0.99 (0.84, 1.19)16.66	0.03 (-94 (0.06,	1.12 (0.03 (-0.11,	0.9473 (0.7144,	1.01 (0.73 (0.4488,	0.98 (0.78, 1.23)
Second trimester	1.05 (0.88, 1.24)26.04	0.08 (-85 (0.01,	0.09 (-0.05, 01.23	0.8584 (0.6452,	1.05 (0.84 (0.5292,	1.00 (0.80, 1.26)
Third trimester	1.06 (0.89,1.26)**38.05**	0.0994 (0.00, 0.1870,	1.28 (0.12 (-0.03,	0.9481 (0.7050,	1.10 (0.81 (0.5096,	0.97 (0.77, 1.22)
Total pregnancy	**28.59**1.04 (0.**32,**	0.07 (-90 (0.02,	1.22 (0.08 (-0.06,	0.9078 (0.6848,	1.06 (0.78 (0.4892,	0.98 (0.78, 1.23)
**PM_{10}_total ($\mu g/m^3$)**						
First trimester	1.01 (0.83,1.23)10.02	0.03 (-88 (0.08,	0.00 (-88 (0.17, 0.1764,	0.8859 (0.6230,	0.5994 (0.3080,	0.88 (0.65, 1.18)
Second trimester	0.90 (0.74,1.10)14.33	0.10 (-80 (0.01,	1.17 (0.08 (-0.11,	0.8073 (0.5539,	1.02 (0.73 (0.3987,	0.95 (0.71, 1.27)
Third trimester	1.05 (0.88, 1.24)22.96	1.09 (0.03 (-0.06,	0.01 (-0**1.46 (1.13,**	0.84 (0.51, 1.09	0.84 (0.51, **1.**38**16**	1.06 (0.83, 1.34)
Total pregnancy	27.38 (-2.31, 571.06	0.09 (-86 (0.01,	1.27 (0.05 (-0.10,	0.8661 (0.6436,	1.07 (0.61 (0.36,	0.94 (0.73, 1.20)

[a]represents the increase in odds of PTB for a per IQR increase in PM_{10} exposure;
[b]represents the increase in odds of TLBW for a per IQR increase in PM_{10} exposure;
[c]represents the increase in odds of SGA for a per IQR increase in PM_{10} exposure

Model adjusted for infant's sex, maternal age, pre-pregnancy maternal body mass index (BMI), maternal educational level, maternal smoking during first 3 months of pregnancy, and environmental tobacco smoke exposure during pregnancy, interaction with maternal education

eTable 3b Association of PM_{10} exposures with birth outcomes stratified by maternal education for outcomes with significant exposure*maternal education interaction term in table 3a

Per IQR increase in exposure	Birth weight, grams (N=7,683)	
	Maternal education: O level or below	Maternal education: A level or above
**PM_{10}_other ($\mu g/m^3$)**		
Third trimester	-6.52 (-25.65, 12.61)	**28.84 (7.91, 49.76)**
Total pregnancy	-0.74 (-19.78, 4.09)	**25.14 (4.62, 45.65)**

Model adjusted for infant's sex, maternal age, pre-pregnancy maternal body mass index (BMI), maternal smoking during first 3 months of pregnancy, and environmental tobacco smoke exposure during pregnancy, stratified by maternal education.

Per IQR increase in exposure	BL, cm -(N=5,037)	
	Maternal education: O level or below	Maternal education: A level or above
**PM_{10}_road ($\mu g/m^3$)**		
First trimester	0.00 (-0.07, 0.07)	**0.12 (0.02, 0.22)**
Second trimester	-0.03 (-0.10, 0.04)	**0.11 (0.01, 0.20)**
Third trimester	-0.01 (-0.08, 0.06)	**0.13 (0.03, 0.22)**
Total pregnancy	-0.01 (-0.09, 0.06)	**0.12 (0.03, 0.23)**

Model adjusted for infant's sex, maternal age, pre-pregnancy maternal body mass index (BMI), maternal smoking during first 3 months of pregnancy, and environmental tobacco smoke exposure during pregnancy, stratified by maternal education

eTable 4. The adjusted associations between PM_{10}_total trimester average exposures (TAEs) and adverse birth outcomes

	Mean difference			Odd ratios		
PM_{10} exposure	Birth weight, grams (95% CI) (N=7,683)	Head circumference, cm (95% CI) (N=6,309)	Birth length, cm (95% CI) (N=5,185)	PTB[a] (95% CI) (N=7,984)	TLBW[b] (95% CI) (N=7,350)	SGA[c] (95% CI) (N=7,493)
PM_{10}_total						
T1	9.18 (-7.62, 25.97)	**0.10 (0.04, 0.15)**	**0.15 (0.07, 0.23)**	0.97 (0.83, 1.14)	0.80 (0.61, 1.07)	0.92 (0.81, 1.05)
T2	5.77 (-11.00,22.53)	-0.03 (-0.08, 0.02)	0.04 (-0.05, 0.13)	**0.83 (0.70, 0.98)**	1.07 (0.81, 1.43)	0.99 (0.87, 1.14)
T3	1.64 (-12.92, 16.21)	0.02 (-0.03, 0.06)	0.03 (-0.04, 0.10)	1.08 (0.94, 1.25)	**1.35 (1.08, 1.68)**	**1.17 (1.04, 1.30)**

Note: Results are taken from mutually adjusted models, in which exposure variables PM_{10}_total TAEs were simultaneously entered in the regression to evaluate their independent effects on adverse birth outcomes, with additional adjustment for gestational weeks (only for birth weight), infant's sex, and maternal age, pre-pregnancy maternal BMI, maternal educational level, maternal smoke during first 3 months of pregnancy and environmental tobacco smoke exposure during pregnancy

[a]represents the increase in odds of PTB for a per IQR increase in PM_{10}_total;

[b]represents the increase in odds of TLBW for a per IQR increase in PM_{10}_total;

[c]represents the increase in odds of SGA for a per IQR increase in PM_{10}_total.

eTable 5 The adjusted associations between PM_{10}_road and PM_{10}_other for same pregnancy period (T1, T2, T3 and WP)

	Mean difference			Odd ratios		
PM_{10} exposure	Birth weight, grams (95% CI) (N=7,683)	Head circumference, cm (95% CI) (N=6,309)	Birth length, cm (95% CI) (N=5,185)	PTB[a] (95% CI) (N=7,984)	TLBW[b] (95% CI) (N=7,350)	SGA[c] (95% CI) (N=7,493)
First trimester						
Road	-3.89 (-17.87, 10.09)	-0.02 (-0.06, 0.03)	0.00 (-0.07, 0.07)	1.01 (0.89, 1.16)	**1.19 (1.00, 1.42)**	1.03 (0.93, 1.14)
Other	16.14 (-0.28, 32.56)	**0.06 (0.02, 0.11)**	**0.10 (0.02, 0.18)**	0.96 (0.82, 1.13)	0.90 (0.69, 1.17)	0.98 (0.86, 1.12)
Second trimester						
Road	-4.57 (-18.36, 9.22)	-0.02 (-0.07, 0.02)	0.00 (-0.07, 0.06)	1.00 (0.88, 1.14)	1.16 (0.97, 1.38)	1.02 (0.92, 1.13)
Other	12.24 (-4.42, 28.90)	0.02 (-0.03, 0.07)	0.05 (-0.03, 0.13)	0.98 (0.84, 1.16)	1.05 (0.81, 1.35)	1.03 (0.91, 1.18)
Third trimester						
Road	-3.18 (-17.03, 10.66)	-0.02 (-0.06, 0.02)	0.01 (-0.05, 0.08)	0.96 (0.83, 1.10)	1.12 (0.94, 1.35)	1.00 (0.91, 1.12)
Other	11.27 (-5.70, 28.25)	0.04 (-0.01, 0.09)	0.06 (-0.03, 0.14)	1.06 (0.90, 1.25)	1.11 (0.86, 1.44)	1.08 (0.95, 1.22)
Whole ~~Preganancy~~Pregnancy						
Road	-4.30 (-18.61, 10.01)	-0.02 (-0.07, 0.02)	0.00 (-0.07, 0.07)	0.99 (0.87, 1.14)	1.17 (0.97, 1.40)	1.02 (0.92, 1.13)
Other	13.75 (-2.91, 30.40)	0.04 (-0.01, 0.09)	0.08 (-0.01, 0.16)	1.00 (0.85, 1.18)	1.02 (0.78, 1.32)	1.03 (0.91, 1.18)

eTable 6. Associations between source-specific PM_{10} exposure in different pregnancy periods and adverse birth outcomes restricting study samples to all women whose gestation finished at 37 weeks

Per IQR increase in exposure	Term low birthweight		Small for gestational age	
	Unadjusted (N=11,302)	Adjusted (N=7,350)	Unadjusted (N=11,302)	Adjusted (N=7,171)
**PM_{10}_road (μg/m^3)**				
First trimester	**1.20 (1.06, 1.30)**	**1.15 (0.99, 1.34)**	1.05 (0.98, 1.13)	1.03 (0.94, 1.13)
Second trimester	**1.18 (1.06, 1.32)**	**1.18 (1.02, 1.37)**	1.06 (0.99, 1.13)	1.05 (0.96, 1.13)
Third trimester	**1.18 (1.06, 1.32)**	**1.17 (1.00, 1.36)**	**1.07 (1.01, 1.15)**	1.06 (0.97, 1.15)
Total pregnancy	**1.20 (1.07, 1.34)**	**1.22 (1.00, 1.38)**	1.07 (1.00, 1.14)	1.05 (0.96, 1.15)
**PM_{10}_other (μg/m^3)**				
First trimester	1.15 (0.99, 1.34)	1.02 (0.82, 1.28)	1.08 (1.00, 1.17)	1.02 (1.00, 1.14)
Second trimester	**1.19 (1.02, 1.39)**	1.16 (0.94, 1.44)	**1.10 (1.02, 1.20)**	1.07 (0.96, 1.19)
Third trimester	**1.23 (1.05, 1.43)**	1.20 (0.97, 1.49)	**1.18 (1.08, 1.27)**	1.11 (1.00, 1.24)
Total pregnancy	**1.20 (1.03, 1.40)**	1.13 (0.91, 1.41)	**1.13 (1.04, 1.22)**	1.07 (0.96, 1.20)
**PM_{10}_total (μg/m^3)**				
First trimester	0.99 (0.82, 1.22)	0.77 (0.58, 1.01)	0.96 (0.87, 1.07)	0.92 (0.80, 1.05)
Second trimester	1.11 (0.91, 1.35)	1.08 (0.83, 1.41)	1.01 (0.91, 1.12)	1.01 (0.88, 1.15)
Third trimester	**1.24 (1.05, 1.46)**	**1.39 (1.12, 1.74)**	**1.20 (1.10, 1.31)**	**1.21 (1.08, 1.36)**
Total pregnancy	**1.20 (1.01, 1.42)**	**1.11 (0.88, 1.40)**	**1.09 (1.00, 1.20)**	1.07 (0.96, 1.21)

Model adjusted for infant's sex, maternal age, pre-pregnancy maternal body mass index (BMI), maternal educational level, maternal smoking during first 3 months of pregnancy, and environmental tobacco smoke exposure during pregnancy

~~eTable 7.~~eTable 7 Associations between source-specific PM_{10} exposure in different pregnancy periods and adverse birth outcomes restricting study samples to first child

Per IQR increase in exposure	Mean difference			Odd ratios		
	Birth weight, grams (N=2,949)	Head Circumference, cm (N=2,450)	Birth length, cm (N=2,087)	PTB[a] Case: 601 (N=2,982)	TLBW[b] Case: 271 (N=2,785)	SGA[c] Case: 928 (N=2,949)
**PM_{10}_road ($\mu g/m^3$)**						
First trimester	5.47 (-13.56, 24.50)	0.02 (-0.04, 0.08)	0.02 (-0.07, 0.11)	0.99 (0.84, 1.17)	1.15 (0.92, 1.43)	0.96 (0.84, 1.10)
Second trimester	4.53 (-14.56, -23.62)	0.00 (-0.06, 0.06)	0.00 (-0.09, 0.09)	0.99 (0.83, 1.17)	1.18 (0.95, 1.47)	1.01 (0.89, 1.15)
Third trimester	6.29 (-12.74, 25.31)	0.02 (-0.04, 0.08)	0.04 (-0.06, 0.13)	0.97 (0.82,1.14)	1.11 (0.88,1.40)	1.00 (0.88,1.13)
Total pregnancy	5.65 (-14.22, 25.51)	0.01 (-0.05, 0.07)	0.02 (-0.07, 0.12)	0.98 (0.83, 1.17)	1.16 (0.92, 1.46)	0.99 (0.87, 1.13)
**PM_{10}_other ($\mu g/m^3$)**						
First trimester	**25.38 (3.70, 47.06)**	0.07 (-0.01, 0.07)	**0.11 (0.01, 0.22)**	0.89 (0.73, 1.08)	0.95 (0.70, 1.29)	0.90 (0.77, 1.05)
Second trimester	18.91 (-3.41, 41.23)	0.01 (-0.04, 0.10)	0.05 (-0.06, 0.16)	0.90 (0.74,1.10)	1.04 (0.77, 1.40)	1.00 (0.86, 1.17)
Third trimester	21.17 (-1.30, 43.64)	0.06 (-0.01, 0.13)	0.10 (-0.01, 0.21)	0.92 (0.75,1.12)	1.06 (0.79, 1.43)	0.98 (0.84, 1.15)
Total pregnancy	**22.97 (0.73, 45.22)**	0.06 (-0.01, 0.13)	0.10 (-0.01, 0.21)	0.90 (0.73, 1.10)	1.01 (0.75, 1.37)	0.96 (0.82, 1.12)
**PM_{10}_total ($\mu g/m^3$)**						
First trimester	16.85 (-9.89, 43.58)	**0.13 (0.04, 0.21)**	**0.16 (0.03, 0.29)**	0.91 (0.72,1.15)	0.76 (0.52, 1.10)	**0.81 (0.67, 0.97)**
Second trimester	10.08 (-18.03, 38.20)	-0.02 (-0.11, -0.07)	-0.04 (-0.18, 0.11)	0.80 (0.63,1.03)	0.90 (0.61,1.33)	1.04 (0.86, 1.26)
Third trimester	-4.78 (-28.35, 18.79)	-0.02 (-0.09, 0.06)	0.02 (-0.09, 0.13)	1.10 (0.90, 1.34)	**1.36 (1.00, 1.84)**	**1.17 (1.00, 1.37)**
Total pregnancy	5.44 (-17.91, 28.78)	0.04 (-0.03,0.11)	0.09 (0.03, 0.20)	0.95 (0.78, 1.17)	0.96 (0.70, 1.32)	1.00 (0.85,1.17)

***: p<0.01, **: p<0.05, *: p<0.1

[a]represents the increase in odds of Preterm birth for a per IQR increase in PM_{10} exposure;

[b]represents the increase in odds of Term low birth weight for a per IQR increase in PM_{10} exposure;

[c]represents the increase in odds of Small for gestational age for a per IQR increase in PM_{10} exposure

Model adjusted for infant's sex, maternal age, pre-pregnancy maternal body mass index (BMI), maternal educational level, maternal smoking during first 3 months of pregnancy, and environmental tobacco smoke exposure during pregnancy

eTable 8. **Comparison of major confounders and outcomes for those with missing and non-missing outcome data and non-missing confounder data**

	Excluded from analyses due to missing birthweight data (n=124)	Included in analyses* (n = 11,896)	Birth weight (grams) Mean ± SD	p-value for difference
Infant's sex				
Male, n (%)	65 (52.42)	6,150 (51.70)	3,467.97 ± 561.73	0.873
Female, n (%)	59 (47.58)	5,746 (48.30)	3,359.16 ± 510.09	
Maternal age, mean ± SD	27.39 ± 5.31	27.79 ± 4.85	-	0.38
Education				0.554
O level or below, n (%)	72 (67.92)	6,914 (65.17)	3414.43 ± 537.13	
A level or above, n (%)	34 (32.08)	3,695 (34.83)	3464.37 ± 507.05	
Missing data	1,305			
Maternal BMI				0.752
BMI<18.5	7 (6.86)	503 (5.10)	3197.76 ± 510.93	
18.5≤BMI<25, n (%)	77 (75.49)	7,282 (73.88)	3415.95 ± 513.41	
25≤BMI<30, n (%)	13 (12.75)	1,524 (15.46)	3513.33 ± 539.53	
BMI≥30, n (%)	5 (4.90)	547 (5.55)	3593.64 ± 622.71	
Missing data	2,062			
Maternal smoking				0.715
Non-smoker	85 (73.91)	8,453 (75.39)	3466.28 ± 527.18	
Smoker	30 (26.09)	2,760 (24.61)	3284.85 ± 549.80	
Missing data	692			
Environmental smoking				0.95
Not exposed	36 (39.13)	3,480 (38.81)	3491.95 ± 502.52	
Exposed	56 (60.87)	5,487 (61.19)	3391.21 ± 526.42	
Missing data	2,961			

*Analysis for birthweight

-	-	Infant's sex			maternal age			maternal education level		
-	-	Male	Female	Chi 2	num. of obs.	mean	t test	O level or below	A level or above	Chi 2
**Birthweight**	missing (n=124)	65 (52.42%)	59 (47.58%)		117	27.39		72 (67.92%)	34 (32.08%)	
	non-missing (n=11,896)	6,150 (51.70%)	5,746 (48.30%)	0.873	11,133	27.79	0.38	6,914 (65.17%)	3,695 (34.83%)	0.554
**Head circumference**	missing (n=2,775)	1,454 (52.40%)	1,321 (47.60%)		**2,515**	**27.62**		**1,505 (67.40%)**	**728 (32.60%)**	-
	non-missing (n=9,245)	4,761 (51.50%)	4,484 (48.50%)	0.406	**8,735**	**27.83**	**0.06**	**5,481 (64.62%)**	**3,001(35.38%)**	**0.014**
**Birth length**	missing (n=4,437)	2,308 (52.02%)	2,129 (47.98%)		**4,084**	**27.65**		**2,525 (67.23%)**	**1,231 (32.77%)**	
	non-missing (n=7,583)	3,907 (51.52%)	3,676 (48.48%)	0.601	**7,166**	**27.86**	**0.03**	**4,461 (64.10%)**	**2,498(35.90%)**	**0.001**
**Term low birth weight**	missing (n=718)	**408 (56.82%)**	**310 (43.18%)**	-	**673**	**27.22**	-	403 (67.85%)	191 (32.15%)	-
	non-missing (n=11,302)	**5,807 (51.38%)**	**5,495 (48.62%)**	**0.005**	**10,577**	**27.82**	**0.002**	6,583 (65.04%)	3,538 (34.83%)	0.163
**Small for gestational age**	missing (n=124)	65 (52.42%)	59 (47.58%)		117	27.39		72 (67.92%)	34 (32.08%)	
	non-missing (n=11,896)	6,150 (51.70%)	5,805(48.29%)	0.873	11,133	27.79	0.38	6,914 (65.17%)	3,695 (34.83%)	0.554

-	-	Maternal BMI					Maternal smoking			Environmental smoking		
-	-	<18.5	18.5≤BMI<25	25≤BMI<30	>=30	chi2	Non-Smoker	Smoker	chi2	Not exposed	Exposed	chi2
**Birthweight**	missing (n=124)	7 (6.86%)	77 (75.49%)	13 (12.75%)	5 (4.90%)		85 (73.91%)	30 (26.09%)		36 (39.13%)	56 (60.87%)	
	non-missing (n=11,896)	503 (5.10%)	7,282(73.88%)	1,524 (15.46%)	547 (5.55%)	0.752	8,453 (75.39%)	2,760 (24.61%)	0.715	3,480 (38.81%)	5,487 (61.19%)	0.95
**Head circumference**	missing (n=2,775)	**119 (5.62%)**	**1,585 (74.91%)**	**318 (15.03%)**	**94 (4.44%)**		**1,848 (73.39%)**	**670 (26.61%)**		750 (38.74%)	1,186 (61.26%)	
	non-missing (n=9,245)	**391 (4.99%)**	**5,774 (73.63%)**	**1,219 (15.54%)**	**458 (5.84%)**	**0.049**	**6,690 (75.94%)**	**2,120 (24.06%)**	**0.009**	2,766 (38.83%)	4,357 (61.17%)	0.941
**Birth length**	missing (n=4,437)	**194 (5.57%)**	**2,613 (75.06%)**	**518 (14.88%)**	**156 (4.48%)**		**3,001 (73.36%)**	**1,090 (26.64%)**		**1,211 (37.37%)**	**2,030 (62.63%)**	
	non-missing (n=7,583)	**316 (4.88%)**	**4,746 (73.27%)**	**1,019 (15.73%)**	**396 (6.11%)**	**0.002**	**5,537 (76.51%)**	**1,700 (23.64%)**	**0.000**	**2,305 (39.62%)**	**3,513 (60.38%)**	**0.035**
**Term low birth weight**	missing (n=718)	**38 (6.73%)**	**405 (71.68%)**	**87 (15.40%)**	**35 (6.19%)**		488 (72.84%)	182 (27.16%)	-	175 (35.35%)	320 (64.65%)	
	non-missing (n=11,302)	**472 (5.03%)**	**6,954 (74.03%)**	**1,450 (15.44%)**	**517 (5.50%)**	**0.002**	8,050 (75.53%)	2,608 (24.47%)	0.116	3,341 (39.01%)	5,223 (60.99%)	0.104
**Small for gestational age**	missing (n=124)	7 (6.86%)	77 (75.49%)	13 (12.75%)	5 (4.90%)		85 (73.91%)	30 (26.09%)		36 (39.13%)	56 (60.87%)	
	non-missing (n=11,896)	503 (5.10%)	7,282(73.88%)	1,524 (15.46%)	547 (5.55%)	0.752	8,453 (75.39%)	2,760 (24.61%)	0.715	3,480 (38.81%)	5,487 (61.19%)	0.95

eTable ~~8~~9. Model checking in multiple imputation: largest fraction of missing information (FMI)

	Birth weight, grams (N=11,896)	Head Circumference, cm (N=9,245)	Birth length, cm (N=7,583)	~~Preterm Birth[a]~~PTB[a] Case: 601 (N=12,020)	~~Term low birthweight[b]~~ TLBW[b] Case: 271 (N=11,302)	~~Small for gestational age[c]~~ SGA[c] Case: 928 (N=11,896)
**PM_{10}_road (μg/m³)**						
First trimester	0.38	0.22	0.30	0.31	0.37	0.40
Second trimester	0.32	0.30	0.23	0.30	0.34	0.37
Third trimester	0.30	0.13	0.23	0.48	0.47	0.37
Total pregnancy	0.25	0.25	0.23	0.26	0.45	0.31
**PM_{10}_other (μg/m³)**						
First trimester	0.23	0.34	0.21	0.25	0.33	0.39
Second trimester	0.35	0.40	0.29	0.34	0.36	0.37
Third trimester	0.40	0.39	0.35	0.35	0.39	0.39
Total pregnancy	0.34	0.27	0.25	0.26	0.30	0.38
**PM_{10}_total (μg/m³)**						
First trimester	0.29	0.28	0.38	0.21	0.29	0.37
Second trimester	0.33	0.39	0.45	0.34	0.47	0.27
Third trimester	0.32	0.41	0.40	0.46	0.34	0.42
Total pregnancy	0.21	0.38	0.27	0.30	0.52	0.25

[a]represents the increase in odds of Preterm birth for a per IQR increase in PM_{10} exposure;

[b]represents the increase in odds of Term low birth weight for a per IQR increase in PM_{10} exposure;

[c]represents the increase in odds of Small for gestational age for a per IQR increase in PM_{10} exposure
